# Metabolic Adaptation of *Paracoccidioides brasiliensis* in Response to *in vitro* Copper Deprivation

**DOI:** 10.3389/fmicb.2020.01834

**Published:** 2020-08-10

**Authors:** Guilherme Petito, Juliana Santana de Curcio, Maristela Pereira, Alexandre Melo Bailão, Juliano Domiraci Paccez, Gabriel Brum Tristão, Camila Oliveira Barbosa de Morais, Marcelo Valle de Souza, Agenor de Castro Moreira Santos, Wagner Fontes, Carlos André Ornelas Ricart, Célia Maria de Almeida Soares

**Affiliations:** ^1^Laboratório de Biologia Molecular, Instituto de Ciências Biológicas, Universidade Federal de Goiás, Goiânia, Brazil; ^2^Programa de Pós-graduação em Genética e Biologia Molecular, Universidade Federal de Goiás, Goiânia, Brazil; ^3^Departamento de Biologia Celular, Instituto de Biologia, Universidade de Brasília, Brasília, Brazil

**Keywords:** copper depletion, pathogenic fungus, proteomic analysis, cell wall, ITRAQ

## Abstract

Copper is an essential micronutrient for the performance of important biochemical processes such as respiration detoxification, and uptake of metals like iron. Studies have shown that copper deprivation is a strategy used by the host against pathogenic fungi such as *Cryptoccocus neoformans* and *Candida albicans* during growth and development of infections in the lungs and kidneys. Although there are some studies, little is known about the impact of copper deprivation in members of the *Paracoccidioides* genus. Therefore, using isobaric tag labeling (iTRAQ)-Based proteomic approach and LC-MS/MS, we analyzed the impact of *in vitro* copper deprivation in the metabolism of *Paracoccidioides brasiliensis*. One hundred and sixty-four (164) differentially abundant proteins were identified when yeast cells were deprived of copper, which affected cellular respiration and detoxification processes. Changes in cellular metabolism such as increased beta oxidation and cell wall remodeling were described.

## Introduction

Based on the ability to cycle between reduced and oxidized states the metal copper is an essential micronutrient to organisms. Copper acts as a cofactor for superoxide dismutase Cu/Zn (Sod1p), an important detoxification enzyme, and cytochrome c oxidase (Coxp), fundamental to the cellular respiration process. Copper in the cupric (Cu^2+^) state is reduced to (Cu^1+^) by iron reductases (Fre1p, Fre2p) in the extracellular medium (Hassett and Kosman, 1995) and transported into cells by high affinity transporters (Ctr1p, Ctr3p, Ctr4p) (Dancis et al., 1994; Waterman et al., 2012). Then, copper is distributed by chaperones as Ccs1p, with an N-terminal domain responsible for transferring the metal to Sod1p. This process is fundamental for the activation of this enzyme (Fukuoka et al., 2017). The Atx1p chaperone delivers copper to a P-type ATPase (Ccc2p) present in the membrane of the Golgi complex (Lin et al., 1997). In *Saccharomyces cerevisiae*, when there is a compromise in the activity of Sod1p, the Atx1p 47 is described as a protein that protects against reactive oxygen species (Lin et al., 1997; Smith et al., 2017). Furthermore, the Cox17p chaperone is an important copper donor to cytochrome c oxidase (Coxp) (Horng et al., 2004), present in the respiratory chain complex IV. The enzyme is a terminal oxidase of the respiratory chain and presents a highly conserved heme-copper catalytic structure (Khalimonchuk and Rödel, 2005).

Since copper is an essential trace element presenting toxic properties when in excess, organisms developed sophisticated mechanisms to provide the micronutrient to biological processes while preventing Cu^1+^ toxicity. This includes the increase in the production of toxic hydroxyl radicals, damaging DNA and proteins (Urbanski and Berêsewicz, 2000; García-Santamarina and Thiele, 2015). Mechanisms that allow copper homeostasis at an appropriate concentration operates at several levels. Copper homeostasis in fungi is obtained at both transcriptional and post-transcriptional levels (Gross et al., 2000). In the model fungus *S. cerevisiae* the cell surface metaloreductases Fre1p and Fre2p reduce Cu^2+^ to Cu^1+^ (Georgatsou et al., 1997). Also, the high-affinity transporters Ctr1p and Ctr3p, regulated at transcriptional level by copper availability, are responsible for the metal uptake (Pena et al., 2000).

Stringent control of Cu uptake is also critical for avoiding excessive intracellular accumulation and toxicity (Peña et al., 1999). Under the exposure to a high-Cu environment, *S. cerevisiae* transcriptional factor MAC1 is rapidly degraded, and that response is accompanied by a decreased expression of the target Ctrp transporters at both the transcriptional and post-translational levels (Zhu et al., 1998). Another transcriptional factor responsive to excessive copper is ACE1; submitted to these conditions this transcriptional factor induces the expression of copper detoxification genes such as *Sod1*, *Cup1*, and *Crs5* (Smith et al., 2017). In *C. albicans* the response to copper excess involves the activation of the copper extrusion pump Crp1p, responsible for transporting copper from the cytoplasm to the extracellular environment coupled to the expression of copper metallothionein Cup1p, which binds copper when at high concentration inside the cells (Weissman et al., 2000).

An important aspect of host-pathogen interaction is the homeostasis of copper during infection. In this sense, the host can sequester copper in a process known as nutritional immunity, which prevents the pathogen from obtaining the metal. For example, the opportunistic fungal pathogen *C. albicans* faces copper nutritional immunity since the calprotectin protein from the host, sequester Cu by binding Cu^2+^ with subpicomolar affinity, inducing copper starvation (Besold et al., 2018). It has also been shown that copper poisoning is used as microbicidal strategy to control bacterial and fungal infections (Mackie et al., 2016; Ladomersky et al., 2017; Wiemann et al., 2017). Infection based studies suggest that macrophages elevate copper levels inside the phagosome by locating the P-type copper ATPase pump (ATP7a) to the phagosome membrane. In addition, such studies also demonstrated that copper detoxifying fungal machinery is required for virulence (Mackie et al., 2016; Wiemann et al., 2017).

Members of the *Paracoccidioides* genus are the causative agents of paracoccidioidomycosis (PCM), a fungal disease highly prevalent in Latin America (Restrepo, 1985; Martinez, 2015). The infection begins after inhalation of conidia or mycelial fragments that reach the alveoli where they differentiate into yeast cells, a process highly dependent on temperature and crucial for the disease establishment (McEwen et al., 1987; San-Blas et al., 2002). Several studies have demonstrated the importance of copper for different organisms (Peña et al., 1999; Gross et al., 2000; Ding et al., 2014a). However, little is known about the impact of this metal deprivation in the genus *Paracoccidioides*. Previous *in silico* analysis have shown that *Paracoccidioides* spp. have homologous copper homeostasis-related proteins such as metalloreductases (Fre1p, Fre3p), high and low affinity copper transporters Ctr3p and Ctr2p respectively, copper metalloregulatory transcription factor (*Mac1*), metallochaperone (Atx1p), transporting P-type ATPase (Ccc2p); and enzymes related to the detoxification process (Silva et al., 2011).

In this work we suggested that *P. brasiliensis* can face copper deprivation upon macrophage infection. We also observed that *in vitro* copper depletion affected the activity of cytochrome c oxidase. The latter is a process accompanied by increase in the expression of the enzyme alternative oxidase (Aoxp), associated with an alternative respiratory pathway. A decrease in the expression of copper dependent Sod1p influenced the detoxification capacity, leading to an increased expression of thioredoxin and superoxide dismutase Mn/Fe (Sod5p). Enzymes of the pentose phosphate pathway which is related to the production of NADPH this being associated with detoxification processes, as well as the chaperone Atx1p, were increased. Up-regulation of proteins of beta-oxidation, the glyoxylate cycle, and cell wall remodeling were also identified.

## Materials and Methods

### Microorganism and Growth Conditions

*P. brasiliensis Pb*18 (ATCC 32069) was employed in this work. The yeast cells were cultivated for 4 days, at 36°C in BHI solid medium supplemented with 4% (w/v) glucose. For the experiment involving copper depletion *P. brasiliensis* yeast cells were incubated in McVeigh/Morton medium (MMcM) (Restrepo and Jimenez, 1980). The copper depleted medium was prepared without CuSO_4_ and supplemented with 50 μM bathocuproinedisulfonic acid (BCS). For control, the medium was supplemented with 10 μM CuSO_4_.

### *P. brasiliensis* Cell Viability Analysis

After the cultivation of yeast cells, viability was determined by membrane integrity analysis using propidium iodide as dead cells marker, as previously described (Grossklaus et al., 2013). Yeast cells (5 × 10^6^ yeast cells/mL) were centrifuged at 3500 × *g* at 4°C for 5 min and the supernatant was discarded. Propidium iodide (1 μg/mL) was added to the cell’s suspension for 20 min in the dark and at room temperature; the cells were analyzed by flow cytometer (BD^®^ Accuri C6 Flow Cytometer). A minimal of 10,000 events per sample was acquired with the FL-3-H channel.

### Macrophage Infection and Generation of Macrophage ATP7a-Silenced Cells

Murine macrophage cell line J774 A. 1 (BCRJ Cell Bank, Rio de Janeiro, accession number 0121) was employed. The macrophages were maintained in RPMI medium (RPMI 1640, Vitrocell, Brazil), the latter being supplemented with non-essential amino acids (Sigma-Aldrich, St. Louis, MO, United States) and 10% (w/v) fetal bovine serum (FBS), at 37°C in 5% CO_2_. 1 × 10^6^ macrophages were seeded into each well of a 24 well tissue plate and IFN-γ (1 U/mL) (Sigma–Aldrich, St. Louis, MO, United States) was added for 24 h at 37°C in 5% CO_2_ for macrophage activation.

ATP7a silenced macrophage cells were generated by transient transfection of a siRNA double-stranded, against the ATP7a gene (Silencer siRNA mouse ATP7a Cat. No. AM16708, Thermo Fisher Scientific, Waltham, MA, United States), using Lipofectamine 2000 as the transfection reagent, following the Lipofectamine 2000 protocol (Invitrogen, Cat. No. 11668-027), in RMPI 1640 medium without any supplementation. A scramble siRNA was used as transfection negative control (Silencer Negative Control Cat. No. AM4611, Thermo Fisher Scientific, Waltham, MA, United States).

Viability of 24, 48, and 96 h macrophages was confirmed by microscopy using Trypan blue dye. Trizol was added in each well, and the total RNA was isolated. RNAs from non-silenced macrophages were obtained as control. After reverse transcription, the ATP7a inhibition was evaluated by qRT-PCR, using the TaqMan gene expression assay (Thermo Fisher Scientific, Waltham, MA, United States; ATP7a TaqMan Cat. No. 437663). The transcript of α-tubulin (TaqMan Cat. No. 492936) was used for normalization of transcript amplification. The best processing time for subsequent transfections was set to 48 h after the quality analysis of 24, 4, and 96 h of ATP7a silencing transcriptional profiles. The Gene ID of these primers are described in Supplementary Table 1.

For fungal burden analysis in the ATP7a silenced macrophages, 5 × 10^6^ cells/mL of silenced and non-silenced macrophages were co-cultivated with 1 × 10^6^ yeast cells/mL in RMPI 1640 medium without supplementation. The cells were co-cultivated for 24 h (Dantas et al., 2009) at 37°C in 5% CO_2_ to allow fungal internalization. Each well was washed twice with 1 mL of PBS 1X to get rid of non-internalized yeast cells. Infected macrophages were lysed with ice-cold ultrapure sterile water, and dilutions of the lysates containing the phagocytized yeast cells were plated in BHI agar and incubated at 37°C in 5% CO_2_ atmosphere. After 7 days of incubation the number of CFU was determined.

### Protein Extraction

Yeast cells cultivated in presence of copper (10 μM CuSO_4_) and absence of this metal (50 μM BCS), were harvested at a 24 h time-point and centrifuged at 5000 × *g*, 4°C for 10 min. The supernatant was discarded, and the cells were washed three times with PBS 1X. Afterward, the cells were resuspended in an extraction buffer containing 20 mM Tris–HCl pH 8.8; 2 mM CaCl_2_ with a mixture of nuclease and protease inhibitors (GE Healthcare). Following this step, the cells were distributed in tubes, glass beads were added, and the cells were disrupted on ice in a bead beater apparatus for 5 cycles of 30 s. Next, the cells were centrifuged at 10,000 *g* for 10 min at 4°C, three times and the quantification of protein extracts was performed as described (Lacerda Pigosso et al., 2017).

### In-Solution Protein Digestion

150 μg of protein extract of each condition/replicate was prepared for trypsin digestion (Queiroz et al., 2014). Ice-cold acetone was added to the protein suspension, followed by incubation at −20°C. After, the samples were centrifuged at 20,000 × *g* for 15 min at 4°C and re-suspended in 8 M urea in 0.05 M triethylammonium bicarbonate buffer (TEAB), pH 7.9. Next, proteins were reduced with 0.005 M DTT for 25 min at 55°C and alkylated with 0.014 M iodoacetamide for 40 min at room temperature, in the dark. Samples were diluted fivefold with 0.001 M CaCl_2_ in 0.025 M TEAB, pH 7.9. Modified trypsin (Promega, Madison, WI, United States) was added in a 1:50 (w/w) substrate ratio. The samples were incubated overnight at 37°C, followed by acidification with 0.1% (v/v) TFA and desalting on homemade C18 microcolumns in P200 low-binding tips. Thereafter, all samples were lyophilized in speed vacuum (Queiroz et al., 2014).

### Isobaric Tag Labeling

Biological triplicates of protein samples from yeast cells were labeled with iTRAQ as previously described (Araújo et al., 2019). Desalting of the samples was performed, and 50 μg of the material was resuspended in 17 μL of 300 mM TEAB. The iTRAQ marker (Reagents Multiplex Kit, Sigma Aldrich) resuspended in 70 μL of ethanol, was added afterward. The samples were incubated for 2 h at room temperature. Equimolar amounts of iTRAQ label were mixed in all samples (copper starvation labeled with 114; control; with 115).

### LC-MS/MS

iTRAQ labeled peptides were analyzed in three technical repetitions each and fractionated using a nano-UHPLC Dionex Ultimate 3000 (Thermo Fisher Scientific) coupled with an Orbitrap EliteTM Hybrid Ion Trap-Orbitrap Mass Spectrometer (Thermo Fisher Scientific) as previously described (Queiroz et al., 2014). Each fraction was loaded onto a pre-column (110 μm × 200 nm) packed in-house with C18 ResiproSilPur of 5 μm with 120 Åpores (Dr. Maisch GmbH, Ammerbuch, Germany). A second chromatography was carried out in column (75 μm × 35 nm) packed in- house with C18 ResiproSilPur of 3 μm and with 120 Åpores (Dr. Maisch GmbH, Ammerbuch, Germany). The process was eluted using a gradient from 100% solvent A [0.1% (v/v) formic acid] and 26% solvent B [0.1% (v/v) formic acid, 95% (v/v) acetonitrile] for 180 min, followed by 26–100% solvent B for 5 min and 100% solvent B for 8 min (a total of 193 min at 200 nL/min). After each run, the column was washed with 90% solvent B and re-equilibrated with solvent A. Mass spectra were acquired in positive ion mode, by applying data-dependent automatic survey MS scan and tandem mass spectra (MS/MS) acquisition modes. Each MS scan in the Orbitrap analyzer (mass range = *m*/*z* 350–1800, resolution = 120,000) was followed by MS/MS of the fifteen most intense ions in the LTQ. Fragmentation in the LTQ was performed by high-energy collision-induced dissociation (HCD), and selected sequenced ions sequences were dynamically excluded every 15 s.

### MS/MS Spectra Processing

Raw data processing used Proteome Discoverer v.1.3 beta (Thermo Fisher Scientific). Searches of the Raw files used Proteome Discoverer with Mascot v.2.3 algorithm against the *P. brasiliensis* database, downloaded using the Database on Demand tool in UniProt/SWISS-PROT^1^ and NCBI^2^ database. For false discovery rates, the number of proteins, protein groups and peptides were filtered to values below 1%. Using Proteome Discoverer two peptides per protein was the minimum value accepted for identification. Raw data was deposited in the Peptide Atlas database^3^ with the following access number PASS01563.

### Data Analysis

The data consisted of three replicates containing global proteome. To increase the reliability, the acceptance criteria were applied, as following: proteins identified with at least two peptides; with high or medium FDR and in at least 2 of 3 replicates. For statistical analysis, unpaired Student’s *t*-test was applied. Statistical difference was set at *p* ≤ 0.05. In this way, two quantitative estimates provided for each protein were utilized: the fold change ratios of differential expression between labeled protein extracts; the *p* value, as cited above. Functional categories were determined by search in Blast2GO platform^4^, Pedant on MIPS-Functional Catalog^5^ and KEGG database^6^. Sequence annotation was assessed using a Blastp algorithm^7^.

### cDNA Synthesis and Quantitative Real Time PCR (RT-qPCR) Analysis

Yeast cells after *in vitro* copper deprivation were harvested at time-points 0, 4, 8, 12, and 24 h. Then, cells were treated with TRIzol (SIGMA-ALDRICH) and the RNA was extracted following the manufacture’s protocol. Then, total RNA was treated with DNase (RQ1 RNase-free DNase, Promega) and subjected to reverse transcription (SuperScript III First-Strand Synthesis SuperMix; Invitrogen, Life Technologies) according to the manufacturer’s recommendation. SYBR green PCR master mix (Applied Biosystems, Foster City, CA, United States) was used in the RT-qPCR assays performed in a Step OnePlus system (Applied Biosystems). Normalization used the gene encoding the L34 protein (PADG_04085). Standard curves were generated by 1:5 dilution of the cDNA, and the relative expression levels of the transcripts were calculated using the standard curve method for relative quantification (Bookout et al., 2006). The used oligonucleotides are described in Supplementary Table 1.

### Analysis of Chitin and Glucan Amount in the Cell Wall of *P. brasiliensis* During *in vitro* Copper Deprivation

Calcoflour White (CFW, Sigma-Aldrich) and Aniline Blue (AB, Sigma-Aldrich) were used to stain *P. brasiliensis* yeast cells to evaluate the effect of copper depletion in the composition of the cell wall, since CFW and AB specifically binds chitin and glucan respectively. *P. brasiliensis* grew in copper depletion or in presence of this metal for 24 h. The cells were stained with the dyes described above and analyzed by fluorescence microscopy as already detailed (De Curcio et al., 2017). In synthesis, for analysis of chitin amount, the cells were fixed in 100% methanol at -80°C for 20 min. The cells were collected, stained with CFW (100 μg/mL in PBS 1 X) for 30 min and washed with PBS 1 X. For analyses of glucan, the cells of *P. brasiliensis* were incubated with aniline blue solution (Sigma) for 5 min and subsequently washed twice with PBS 1 X. Both cells stained with CFW and aniline blue were visualized in a fluorescence microscope (Zeiss Axiocam MRc-Scope A1). The minimum of 50 cells for each microscope slides, in triplicates, were used to evaluate fluorescence intensity of the cells. The software provided the fluorescence intensity (in pixels) and the standard error of each analysis. Statistical comparisons were performed using the Student’s *t*-test and *p* ≤ 0.05 were considered statistically significant.

### Analysis of Mitochondrial Activity of *P. brasiliensis* Exposed to *in vitro* Copper Deprivation

The mitochondrial activity was monitored using the Rhodamine 123 (Sigma-Aldrich) and Mitotracker Green FM (Sigma-Aldrich) fluorescent dyes. Yeast cells were stained according to the manufacturer’s instructions. Initially, yeast cells were harvested by centrifugation of 8000 × *g* for 10 min at 4°C, diluted in PBS 1X to 10^6^ cells/mL and stained during 45 min at 37°C with Mitotracker (400nM). Then, the cells were washed with PBS 1 X and labeled with Rhodamine (2.4 μM) for 45 min at 37°C. Afterward, the cells were washed three times with PBS 1X and analyzed by fluorescence microscopy (Zeiss Axiocam MRc – Scope A1) at 450–490 nm for Mitotracker (FS09) and 515–575 nm filter (FS15) for Rhodamine. All experiments were performed in triplicate and the minimum of 50 cells for each microscope slides were assessed to measure of the fluorescence intensity (in pixels). Student’s *t-*test and *p* ≤ 0.05 were considered statistically significant as described by Chaves et al. (2019).

### Reactive Oxygen Species Evaluation

The generation of reactive oxygen species (ROS) was evaluated using dichlorofluorescein 2′, 7′-diacetate (DCFDA) (Sigma-Aldrich) as described (Ikeda and Sawamura, 2008). *P. brasiliensis* yeast cells were cultivated in presence or absence of copper and collected at 12 and 24 h of treatment. The cells were harvested by centrifugation of 2000 *g* for 5 min at 4°C, diluted in PBS 1X to 10^6^ cells/mL. Then, 1 mL was transferred to a clean eppendorf, added of 1 μL of DCFDA and the mixture could stand for 30 min, in a dark environment. The cells were washed twice with PBS 1X and observed under fluorescence microscopy (Zeiss Axiocam MRc – Scope A1) using the 490–516 nm filter (FS09).

### Enzymatic Activities and Biochemical Tests

For confirmation of differential regulation of some proteins, enzymatic activities and biochemical tests were performed. Cytochrome C oxidase (CCO) activity was performed employing the Cytochrome C Oxidase Assay Kit (CYTOCOX1-Sigma Aldrich) following the manufacturer’s instructions. The CCO activity was evaluated by a colorimetric assay based on observation of the decrease in absorbance of ferrocytochrome C at 550 nm caused by its oxidation to ferricytochrome C by CCO. Triplicates were obtained for each condition. The results were considered statistically significant at *p* ≤ 0.01 by Student’s *t*-test.

Free thiol levels were determined using the Ellman’s reagent, 5, 5′-dithio-bis-(2-nitrobenzoic acid) (DTNB – Sigma Aldrich). A total of 10^6^ yeast cells/mL incubated in the presence or absence of copper, were centrifuged for 5 min at 8000 × *g* and lysed by the addition of 0.5 mL lysis buffer [50 mM Tris-Cl, 150 mM NaCl, 50 mM ethylenediaminetetraacetic acid (EDTA), pH 7.2] and glass beads in equal volume of the cellular pellet. The cells were disrupted on ice in a bead beater apparatus for 5 cycles of 30 s. After centrifugation, 100 μL of the supernatant was added to 100 μL of 500 mM phosphate buffer, pH 7.5, followed by the addition of 20 mL of 1 mM DTNB. Absorbance was measured at 412 nm using a plate reader. Triplicates were obtained for each condition, as previously described (Lacerda Pigosso et al., 2017). Results were considered statistically significant at *p* ≤ 0.01 by Student’s *t*-test.

### Obtaining the Recombinant Protein Atxp and Polyclonal Antibodies Production

The vector pET32a:Pb18Atx1p was synthesized by GenOne (Rio de Janeiro, Brazil). Briefly, the *Atx1* sequence from *P. brasiliensis* (PADG_02352) was codon optimized for *Escherichia coli*, and cloned into *Bam*HI restriction site from vector pET32a (Novagen, Merck-Millipore). For bacterial transformation, 150 ng of DNA from plasmid pET32a:Pb18Atx1p was added to the *E. coli* BL21 competent cell suspension and kept on ice for 1 min. The cells were transferred to an electroporation cuvette and subject to 1800 -Volt electric field to allow entry of the plasmid into the cell. After electroporation, cells were resuspended in 900 μL Luria-Bertani (LB) medium and incubated at 37°C while shaking for 1 h. Aliquots were plated in LB medium containing 100 μg/mL ampicillin for selection of the transformed bacteria. Recombinant protein induction was performed by addition of Isopropyl-β-D thiogalactopyranoside (IPTG) at 0.1 mM. Protein extracts were submitted to 12% SDS-PAGE followed by coomassie blue staining. The bacteria lysis was performed by adding 500 μg/mL lysozyme for 1 h, under constant stirring, at room temperature and sonication in 5 pulses of 10 min. The supernatant from the solubilization process was used for purification of the recombinant Atx1p by nickel column affinity chromatography.

Purified *P. brasiliensis* recombinant Atx1p was inoculated into 6–8-week-old male BALB/c mice to produce polyclonal antibodies. The purified protein was cut from the polyacrylamide gel, macerated in grinder with 2 mL of PBS 1 X, and 200 μL of the solution were applied to the mice. Three immunizations were performed, 15 days apart, intraperitoneally. Fifteen days after the last immunization, the animals were anesthetized with sodium thiopental (40 mg/kg) and lidocaine hydrochloride (2 mg/kg), and blood puncture was performed. The animals were then euthanized by cervical dislocation. Sera collected from unimmunized mice were used as negative control (preimmune). All procedures were approved by the Animal Use Ethics Committee of the Federal University of Goiás (CEUA-PRPI-UFG), under protocol number 092/17.

### Western Blotting and Immunofluorescence Assays

Proteins in SDS-PAGE were transferred to a nitrocellulose membrane that was then incubated with polyclonal anti-Atx1p antibodies at 1:2000 dilution, for 2 h at room temperature. After washing, the membranes were incubated with peroxidase-coupled mouse anti-IgG secondary antibody (1:1000 dilution). The reaction was revealed by chemiluminescence with the ECL Western Blotting substrate kit (GE Healthcare). Negative control was obtained with preimmune mice serum (1: 500 dilution).

For immunofluorescence, 10^6^ yeast cells/mL were fixed in ice cold pure methanol for 3 h at 20°C. After washing with PBS 1 X, part of the fixed cells was permeabilized with 0.25% triton X-100 (Sigma-Aldrich) for 20 min. Subsequently, cells were incubated for 30 min at room temperature, in the dark in blocking buffer containing 3% (w/v) bovine serum albumin (BSA-Sigma), 0.2% (v/v), tween 20 in PBS, followed by incubation with the primary anti-Atx1p polyclonal antibodies at 1:500 dilution, for 1 h. Subsequently there is an addition of fluorescein isothiocyanate-labeled mouse secondary antibody- FITC (Sigma) at 1:750 dilution, for 1 h. Cells were washed three times with PBS 1 X. Images were taken in bright field and at 450–490 nm for visualization of FITC fluorophore, using the Axio Scope A1 fluorescence microscope. Digital images were acquired using AxionVision software (Carl Zeiss AG, Germany).

## Results

### *P. brasiliensis* Survival in ATP7a Silenced Macrophages

**FIGURE 1:**
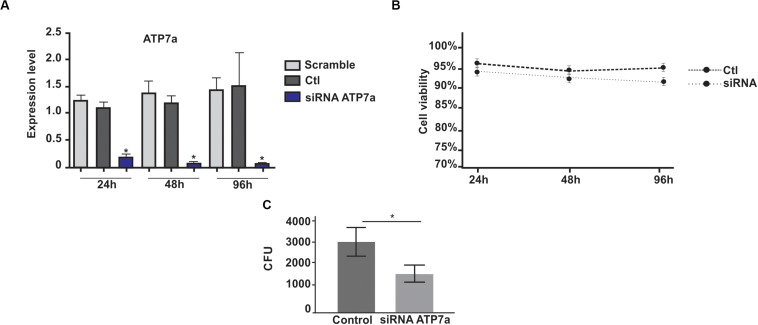
Recovering of *P. brasiliensis* yeast cells after 24 h infection in silenced ATP7a macrophages. **(A)** Transcription levels of ATP7a after 24, 48, and 96 h of transfection with ATP7a siRNAs. Scramble – macrophages transfected with a scramble RNA; Ctl – non- transfected macrophages; siRNA – ATP7a- silenced macrophages. **(B)** Viability of macrophage cells silenced for ATP7a. **(C)** The cells of *P. brasiliensis* (*Pb*18) were used for infection during 24 h in wild type macrophages or silenced for ATP7a. After the infection period, the yeast cells were recovered from both macrophages and the number of fungi was determined by counting Colony Forming Units (CFU). Data are expressed as mean ± standard error (represented using error bars). A statistically significant difference was determined by Student’s *t*-test, (*) represents *p* ≤ 0.05. Control (non-silenced macrophages), SiRNA for ATP7a protein (ATP7a silenced macrophages).

To investigate the influence of copper on the survival of *P. brasiliensis* inside J774A.1 macrophage, we knocked down the P-type copper ATPase pump (ATP7a) of this defense cell. This protein is responsible for translocation of copper from the Golgi to phagolysosome during infection (White et al., 2009). ATP7a silenced J774A.1 macrophage cell line was obtained. The transfection of host cells with siRNAs against ATP7a transcripts promoted a 15 times reduction in copper ATPase mRNA levels (Figure 1A) and such treatment did not affect macrophage viability (Figure 1B). The data analyzes demonstrates that in ATP7a-silenced macrophages, the number of recovered CFU from *P. brasiliensis* was significantly reduced when compared to control macrophages (Figure 1C).

### Fungal Viability and Analysis of Expression of the Copper Transporter Ctr3 Transcript in Response to *in vitro* Copper Deprivation

**FIGURE 2:**
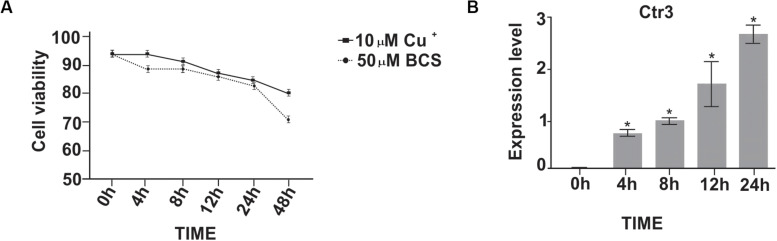
Viability of *P. brasiliensis* at different time points of copper deprivation and expression analysis of *Ctr3* gene. **(A)** The viability of yeast cells of *P. brasiliensis* at time-points of copper deprivation was analyzed. The yeast cells were copper deprived by the addition of 50 μM bathocuproinedisulfonic (BCS); 10 μM of CuSO_4_ was added to control. The viability was determined using flow cytometer (BD^®^ Accuri C6 Flow Cytometer) and at least 10,000 events were analyzed. **(B)** Expression analysis of the *Ctr3* gene in yeast cells during copper deprivation: Relative levels of expression of the *Ctr3* gene (Genbank PADG_05084) after treatment with 50 μM BCS at different time-points. Gene expression values were normalized using the expression values of the transcript coding for L34 (GenBank accession number (PADG_04085) Data are expressed as mean ± standard deviation from triplicates. *Statistically significant (*p* < 0.05).

To determine the impact of copper deprivation on fungal viability, flow cytometry was employed. The results demonstrated that, although the fungus was deprived of copper, cellular viability was not affected within the first 24 h (Figure 2A). After 48 h of growth in MMcM medium there was a decrease in the fungus viability, probably due to the consumption of nutrients in the liquid medium after several h of cultivation. The expression profile of the gene encoding the high affinity copper transport Ctr3p in *P. brasiliensis* was evaluated at different times of copper deprivation. After 24 h of deprivation high induction of *Ctr3* was observed (Figure 2B), indicating that the fungus was in copper deprivation.

### Proteomic Analysis of Yeast Cells Submitted to *in vitro* Copper Deprivation

Proteomic response of yeasts cells subjected to copper deprivation was performed. In this work the iTRAQ method was used to perform proteomic analysis. This method allows to analyze simultaneously multiple samples and has been extensively used in innumerous quantitative proteomics studies (Ruppen et al., 2010). Thus, isobaric tag labeling- iTRAQ permitted us to perform the quantitative comparison of *P. brasiliensis* proteome in two different conditions. Using this method, a total of 164 proteins were differentially expressed in copper deprivation, considering *p* ≤ 0.05 and were categorized according to their cellular functions using the Functional Catalog (FunCat2). The main biological processes regulated by copper deprivation include metabolism (22%), energy (17%), and protein fate (15%) (Supplementary Figure 1). Among the differentially expressed proteins, 155 increased in abundance (Supplementary Table 2) and 09 decreased in abundance (Supplementary Table 3).

### Beta Oxidation and Glyoxylate Cycle Increase in Yeast Cells During *in vitro* Copper Deprivation

**FIGURE 3:**
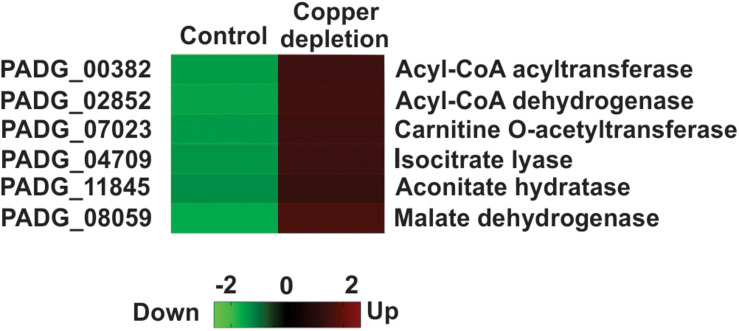
Metabolic pathways regulated in *P. brasiliensis* in response to *in vitro* copper deprivation. Heat map showing increased quantity of proteins from beta oxidation, TCA and glyoxylate cycle in copper deprived *P. brasiliensis* yeast cells. Colors on the heat map indicate differential accumulation; red increased and green decreased. The heat map was constructed by GraphPad Prism software. Copper deprivation (50 μM BCS) and control (10 μM of CuSO_4_).

Proteins related to the pathways, as follows: beta-oxidation: acetyl-CoA acyltransferase (PADG_00382 and PADG_01687), acyl-CoA dehydrogenase (PADG_02852), carnitine O-acetyltransferase (PADG_07023); to the glyoxylate cycle: isocitrate lyase (PADG_04709), aconitate hydratase (PADG_11845) malate dehydrogenase (PADG_08059), and to the TCA cycle: isocitrate dehydrogenase (PADG_04249) were increased, strongly connecting beta oxidation and the glyoxylate cycle in copper-deprived yeast cells (Figure 3).

### Oxidative Stress Response Increases in Yeast Cells During *in vitro* Copper Deprivation, Due to Accumulation of ROS

**FIGURE 4:**
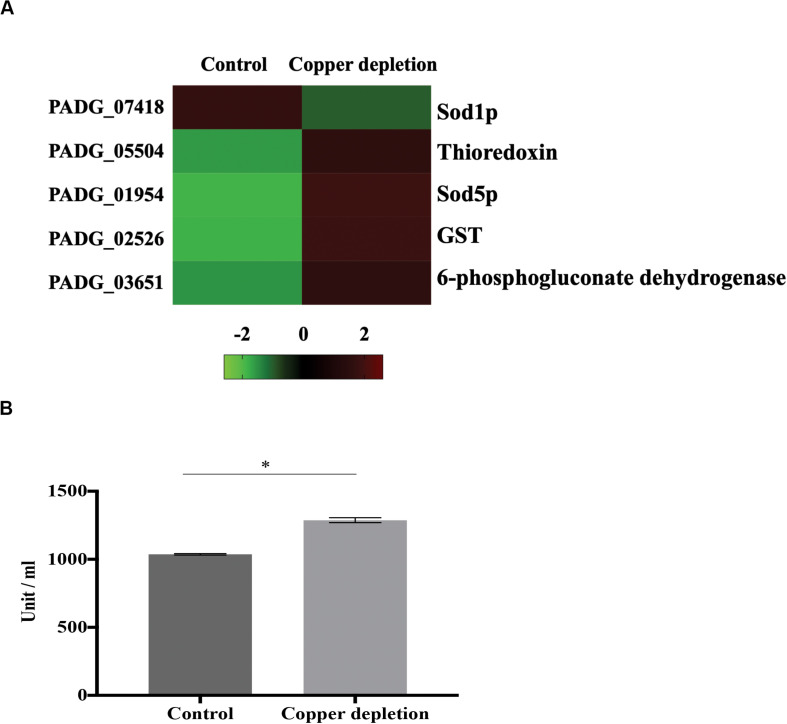
*P. brasiliensis* activates the response to oxidative stress during *in vitro* copper deprivation **(A)** Proteins involved with response to oxidative stress were increased during this metal deprivation. The proteins involved with this response are demonstrated on the heat map; with increased proteins indicated by red color and decreased by green color. Only Sod1p decreased by copper deprivation. **(B)** To determine the enzymatic activity of thioredoxin, yeast cells were treated with 50 μM BCS or 10 μM of CuSO_4_ (control) for 24 h The data were obtained by determining the thiol reduction level in yeast cells, cultivated in the presence or absence of copper. The absorbance was measured at 412 nm. Data are expressed as mean ± standard error (represented using error bars), (^∗^) represents *p* ≤ 0.05.

**FIGURE 5:**
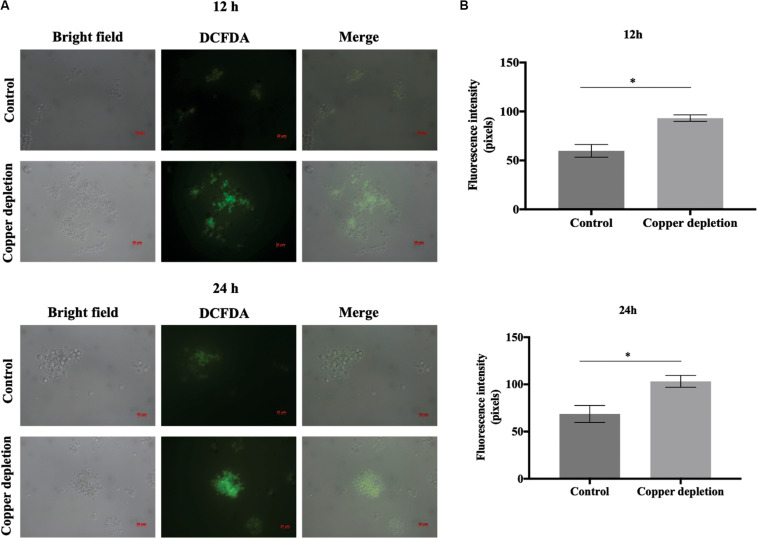
Copper deprivation results in an increase of reactive oxygen species (ROS) **(A)** Detection of ROS during 12 and 24 h of copper deprivation Yeast cells were grown with and without copper, labeled with dichlorofluorescein 2′, 7′-diacetate (DCFDA) and analyzed by fluorescence microscopy. 50 μM BCS indicated copper depletion; control: 10 μM CuSO_4_. Bars indicate the standard deviation **(B)** Fluorescence intensity graphs. The minimum quantity of 50 cells per triplicate were used to construct the graphs. All representative images were magnified 400x. The data for the fluorescence intensity evaluation were obtained through the AxioVision Software (Carl Zeiss). *This demonstrates a significant difference between the samples with the *p*-value of ≤ 0.05. The error bars represent the standard deviation of the samples in triplicates. Control (10 μM of CuSO_4_) and copper deprivation (50 μM BCS).

An important enzyme related to the detoxification of *P. brasiliensis*, Zn/Cu superoxide dismutase (Sod1p) (PADG_07418), was decreased. On the other hand, the following were accumulated: thioredoxin (PADG_05504), Mn/Fe superoxide dismutase (Sod5p) (PADG_01954) and glutathione-S-transferase (GST) (PADG_02526). This suggests that copper deprivation promoted an increase in the formation of reactive radicals. Furthermore, we identified increased levels of 6-phosphogluconate dehydrogenase (PADG_03651), enzyme of the phosphate pentose pathway (Figure 4A). This may be related to the production of NADPH, an important reducing agent that increases the efficiency of detoxification enzymes. A decrease abundance of Sod1p, as well as an increase of detoxification enzymes such as thioredoxin, GSTp and Sod5p suggest that copper deprivation has affected the control of free radicals in the cell. The evaluation of thioredoxin provided increased enzyme activity during copper deprivation (Figure 4B). In addition, ROS detection by fluorescence microscopy using 2,7 dichlorofluorescein marker s, when compared to control, demonstrated increase of ROS after 12 and 24 h in copper-deprived yeast cells compared to control (Figure 5). In this way, data suggest that copper deprivation induced oxidative stress in yeast cells of *P. brasiliensis*, which account for the increase in the cell’s response.

### The Metabolism of the Cell Wall Increases During *in vitro* Copper Deprivation

**FIGURE 6:**
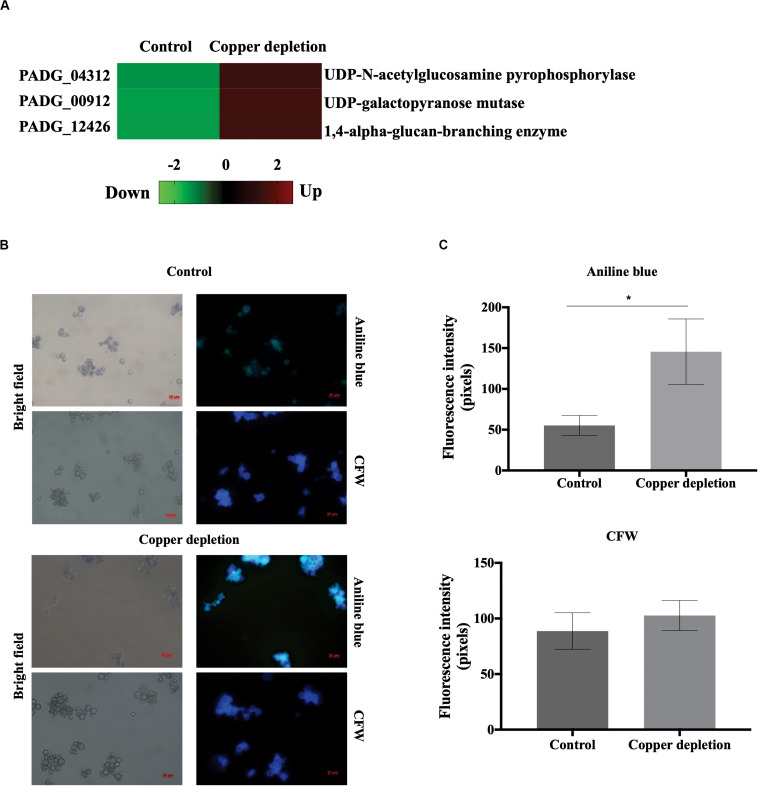
Evaluation of the cell wall metabolism in the yeast cells of *P. brasiliensis* in copper deprivation. **(A)** Heat map showing increased accumulation of proteins related to the cell wall metabolism during copper deprivation, with increased (red color) and decreased (green color) proteins. **(B)** Yeast cells were incubated in medium containing 10 μM of CuSO_4_ (control) or 50 μM BCS (copper depletion) during 24 h. For analysis of the amount of glucan and chitin the yeast cells were stained with aniline blue and Calcofluor White (CFW) respectively and analyzed by fluorescence microscopy. Bars represent the standard deviation. **(C)** Fluorescence intensity in control and copper-deprived yeast cells, both labeled with aniline blue and CFW. To determine significant differences in each condition, pixels intensity values were obtained from triplicates from at least 50 cells, each *indicated with *p-*value of ≤ 0.05. All representative images were magnified 400x, control (10 μM of CuSO_4_) and copper deprivation (50 μM BCS).

Enzymes involved in cell wall synthesis, UDP-N-acetylglucosamine pyrophosphorylase (PADG_04312), UDP-galactopyranose mutase (PADG_00912) and 1,4-alpha-glucan branching enzyme (PADG_12426), increased in the copper-deprived yeast cells (Figure 6A). The images shown in Figure 6B represent an increased fluorescence of yeast cells in copper deprivation when stained with the glucan-binding dye, aniline blue. The fluorescence of chitin was similar in yeast cells that were either deprived, or not, of copper. In this sense, fluorescence intensity quantification confirms that only the amount of glucan increased (Figure 6C). Possibly glucose produced by gluconeogenesis could be used to produce polymers involved in the remodeling the fungal cell wall.

## *In Vitro* Copper Deprivation Induces Alternative Oxidase and Decreased Mitochondrial Activity

**FIGURE 7:**
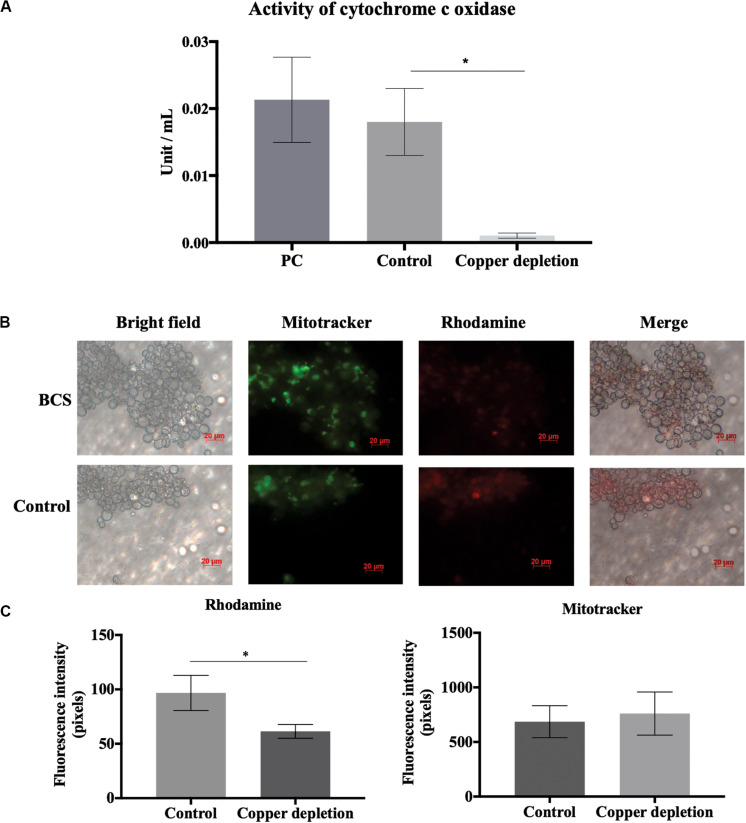
Activity of cytochrome c oxidase (Coxp) and oxidative phosphorylation are regulated by copper deprivation. **(A)** Yeast cells of *P. brasiliensis* were incubated in the presence and absence of copper, and the enzymatic activity of Coxp was evaluated. PC represents the positive control of the Assay Kit (Sigma-Aldrich – CYTOCOX1). Bars represent the standard deviation. **(B)** Yeast cells of *P. brasiliensis* were labeled with mitotracker and rhodamine for verification of integrity and mitochondrial activity, respectively, by fluorescence microscopy. **(C)** Fluorescence intensity graph of yeast cells labeled with mitotracker and rhodamine. The data for fluorescence intensity evaluation were obtained through the AxioVision Software (Carl Zeiss). The values of fluorescence intensity (in pixels) and the standard error of each analysis were used to plot the graph. Data are expressed as mean ± standard error (represented using error bars), (^∗^) representing *p* ≤ 0.05. All representative images were magnified 400x, copper deprivation (50 μM BCS) and control (10 μM of CuSO_4_).

The enzyme alternative oxidase (Aoxp) (PADG_03747) increased in yeast cells when in copper deprivation (Supplementary Table 2). Aoxp is accumulated when there is a compromise in activity of cytochrome c oxidase (Coxp) activity (Horne et al., 2001). Copper is a necessary cofactor for Coxp activity. In this context, the absence of copper could activate the alternative system, mediated by Aoxp, to compensate for a possible loss of Coxp activity due to the reduction of copper levels. In fact, alternative oxidase (PADG_03747) accumulated. Considering the important role of copper in Coxp activity, we hypothesized that the increase in Aoxp accumulation should be related to decreased Coxp activity caused by copper deprivation. In this way, two strategies were performed. The first analysis was to evaluate the enzymatic activity of Coxp. When in comparison to control, activity of Coxp reduced in copper deprivation (Figure 7A). Then, we evaluated the activity of the electron transport chain, by fluorescence microscopy. Analysis of rhodamine fluorescence demonstrated mitochondrial electron transport chain repression in copper depletion (Figures 7B,C). On the other hand, mitochondrial integrity was not compromised, since mitotracker labeling did not show significant differences between treated and control (Figures 7B,C). This data corroborates that copper deprivation could repress electron transport chain, without affecting the integrity of mitochondria.

### The Cytoplasmic Atx1p Copper Chaperone Is Induced in Copper Deprivation: Additional Confirmatory Assays

**FIGURE 8:**
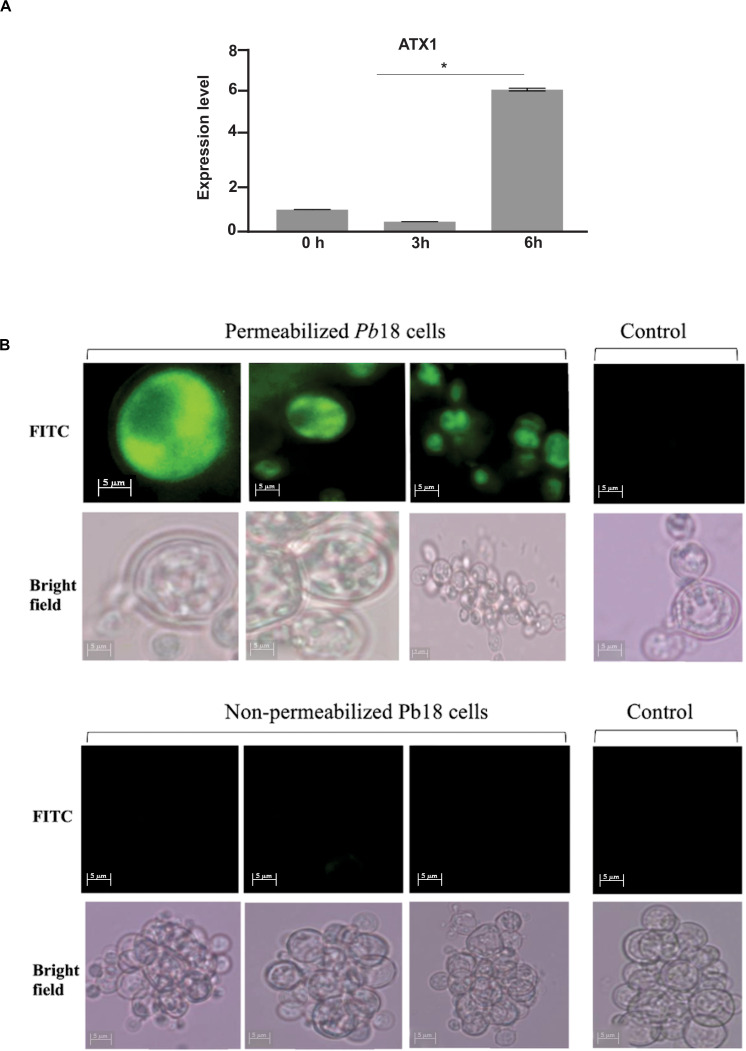
Expression of the transcript and cell localization of Atx1p in *P. brasiliensis***. (A)** Expression analysis of *Atx1* in yeast cells during copper deprivation. Relative levels of expression of the *Atx1* gene in treatment with 50 mM BCS at different time points. Gene expression values were normalized using the expression values of the transcript coding for L34 (GenBank accession number (PADG_04085). *Statistically significant (*p* < 0.05). **(B)** Atx1p immunofluorescence. The immunofluorescence assay was performed with triton X-100 permeabilized and non-permeabilized cells, incubated with anti-Atx1p polyclonal antibodies for 1 h, followed by incubation with the isothiocyanate-labeled mouse anti-IgG secondary antibody fluorescein – FITC for 1 h. Fluorescence was only observed in permeabilized cells. Images were taken in bright field and at 450–490 nm wavelength for viewing FITC fluorophore at 1000x magnification using the Axio Scope A1 fluorescence microscope. Digital images were acquired using AxionVision.

Proteomic analysis demonstrated that the expression of a copper chaperone Atx1p (PADG_02352), increased in copper deprivation (Supplementary Table 2). Studies described that Atx1p plays a critical role in detoxification of reactive oxygen species, which increased in *P. brasiliensis* during *in vitro* copper depletion. In view of this affirmative we performed gene expression analysis by qRT-PCR, which confirmed proteomic data (Figure 8A). Using polyclonal anti-Atx1p antibodies (Supplementary Figure 2) immunofluorescence of Atx1p in *P. brasiliensis* yeast cells was performed. Fluorescence was observed only when permeabilizing the cell, indicating that Atx1p presents cytoplasmic localization in *P. brasiliensis* (Figure 8B).

## Discussion

During host infection, pathogenic fungi may find different copper concentrations on dependence of the infected tissue. *C. neoformans* finds high levels of copper in the lungs at the onset of infection, whereas reduced levels are found during brain infection (Sun et al., 2014). In our study, we infected macrophages silenced for expression of type P copper ATPase (ATP7a) which pumps copper into the phagolysosome. After CFU counting, we noticed, compared to non-silenced macrophages, a lower fungal recovery. Copper accumulation inside macrophages is a strategy used to limit the growth of fungi such as *Aspergillus fumigatus* (Song et al., 2019). In this sense, *A. fumigatus* had an increased survival during infection in ATP7a-deficient zebrafish (Wiemann et al., 2017). In contrast, the data obtained in this work suggest that *P. brasiliensis* is deprived of copper when inside macrophages and that silencing of ATP7a enhanced this deprivation, further compromising the fungus survival. This finding is very surprising since is not in agreement with previous published data for microbial invaders (White et al., 2009; Ladomersky et al., 2017; Wiemann et al., 2017). The reduction on fungal survival observed upon ATP7a silencing could be a result of an intensified copper limiting environment. In agreement we have previously demonstrated that expression of *Crt3* gene is increased in yeast cells infecting macrophages (Dantas et al., 2009). We cannot exclude a hypothetical decreased phagocytosis ability of silenced macrophages. The latter hypothesis is less likely since decreased levels of ATP7a do not cause unrelated effects in macrophages (White et al., 2009). However, additional experiments should be performed to clarify this fact.

Copper is a micronutrient necessary for the activity of enzymes that participate in important processes. These important processes are related to detoxification (Peña et al., 1998), respiration (Horng et al., 2004) and capture of metals (Georgatsou et al., 1997). Therefore, the absence of copper may compromise the fungus’ ability to adapt, survive and infect the host (Ding et al., 2014b; De Oliveira et al., 2014; Broxton and Culotta, 2016). Through a proteomic approach, we identified differentially accumulated proteins related to beta oxidation, glyoxylate cycle, pentose phosphate pathway and cell wall polymer synthesis, as well as others compromised with cellular respiration and detoxification.

Metabolic reprogramming by activation of alternative pathways of carbon consumption is a mechanism characterized in *P. brasiliensis*; this way it can survive in hostile conditions imposed by the host (Parente-Rocha et al., 2015; Chaves et al., 2019). Our data suggest that in copper deprivation occurs a shift of metabolism toward beta oxidation and glyoxylate cycle. This is due to a significant increase in enzymes. In this sense, during the infection of macrophages by *P. brasiliensis*, glucose availability is low and, for the survival of this fungus, oxidation of fatty acids is its energy source (Parente-Rocha et al., 2015). In interferon-gamma primate or non- macrophages, activation of the glyoxylate cycle, is a common strategy used by this fungus (Chaves et al., 2019). The gene induction o of the glyoxylate cycle and beta-oxidation is the strategy employed by *P. brasiliensis* during infection in mouse lung; this is also described during the infection of *A. fumigatus* conidia in neutrophils (Sugui et al., 2008; Lacerda Pigosso et al., 2017). Therefore, the activation of those alternative pathways for energy production could be a mechanism used for *P. brasiliensis* when in response to copper deprivation *in vitro* and *in vivo*.

In view of the protein identification related to cell wall remodeling- the UDP-N-acetylglucosamine pyrophosphorylase (PADG_4312), the UDP-galactopyranose mutase (PADG_00912) and the 1,4-alpha-glucan-branching enzyme (PADG_12426)- we suggest that cell wall remodeling could occur in *P. brasiliensis* when in copper deprivation. In fact, by fluorescence microscopy, we identified an increase of glucans in the cell wall of copper-deprived *P. brasiliensis*. The remodeling of the cell wall is also described during the infection of macrophages by *P. brasiliensis*, in which there is an increase of the glucan and chitin synthesis. This can allow for the multiplication and fungal growth within these defense cells (Chaves et al., 2019). Interestingly, in copper depletion, the synthesis of cell wall polymers was increased, and the glucose produced through the intermediates of glyoxylate cycle could be used to remodel the cell wall.

The ability of *P. brasiliensis* in copper depletion to defend against reactive oxygen species seems affected in a critical way. Indeed, our results show that there was an increase in reactive oxygen species in 12 and 24 h of copper deprivation. Members of the *Paracoccidioides* genus present six isoforms of superoxide dismutases (Sod1-6) in their genomes (Tamayo et al., 2016). Sods 1, 3, and 4 present Cu/Zn domains. Sod3p is extracellularly located and Sod4p is cytoplasmic and both present low level of transcripts. The other Sods 2, 5, and 6 present Fe/Mn domains. The isoforms 2 and 5 are mitochondrial, while Sod6p is cytosolic. All the isoforms transcripts are poorly expressed in mycelia and yeast cells in the genus *Paracoccidioides* (Tamayo et al., 2016). In our analysis Sod1 decreased and Sod 5 increased in yeast cells during copper depletion, accounting for Cu/Zn domains in Sod1p. Additionally, thioredoxin and GST increased in abundance in copper depletion.

The 6-phosphogluconate dehydrogenase (PADG_03651) related to the production of NADPH via the pentose phosphate pathway also accumulated. NADPH plays an important role in maintaining yeast cell antioxidant activity (Kim et al., 2011). Fungi such as *P. lutzii* and *S. cerevisiae*, under oxidative stress requires NADPH, produced by the pentose phosphate pathway, due to its reducing power (Kim et al., 2011; Grossklaus et al., 2013). *Pseudomonas fluorescens*, requires NADPH when the organism undergoes oxidative stress (Singh et al., 2007).

We also identified increase in the chaperone Atx1p in proteomic analyzes. In *S. cerevisiae* Atx1p is a cytosolic protein and facilitates the delivery of copper of the cell surface copper transporter (Ctr1p) to Ccc2p and Fet3p in the secretory pathway (Lin and Culotta, 1995). Therefore, the location of this chaperone in *P. brasiliensis* was analyzed since the compartment in which Atx1p is described directly influences the delivery of intracellular copper, and consequently the availability of this micronutrient inside the cell, influence on the expression of genes and proteins. In addition, Atx1p also may act in the response to oxidative stress, and presents important role in *S. cerevisiae*, protecting the fungal cell against superoxide anion and hydrogen peroxide toxicity (Lin and Culotta, 1995; Lin et al., 1997).

Although there was a significant increase in some proteins of the respiratory chain, we detected by biochemical assays, that Cox1p activity was strongly reduced during copper deprivation. The role of copper as a cofactor of the enzyme Coxp indicates that changes in the levels of this micronutrient might affect the respiratory process. The presence of this metal ensures the transport of electrons and the reduction of molecular oxygen to water from the respiratory chain complex IV (Horne et al., 2001). In *S. cerevisiae*, copper depletion caused by deletion of the *Mac1* results in reduction of the fungus respiratory capacity (Jungmann et al., 1993). Additionally, an alternative oxidase Aoxp was increased in this work. These results suggest that copper deprivation led to impaired respiratory activity in *P. brasiliensis* and that positive Aoxp regulation would compensate for this loss. This enzyme is described in plants and fungi as an important component acting as an alternative respiratory route during impairment in Coxp activity (Horne et al., 2001; Vishwakarma et al., 2015). The ascomycete *Podospora anserina* shows induction of an alternative pathway, mediated by the alternative oxidase when in copper deprivation (Borghouts et al., 2002).

Aoxp also functions as a detoxifying agent in mitochondria in fungi, as well as in the *Paracoccidioides* genus (Hernández et al., 2015; Smith et al., 2017). A relationship between efficiency of the respiratory process, production of ROS and role of superoxide dismutase and Aoxp was described (Broxton and Culotta, 2016). Aoxp contributes to the adaptation of *C. albicans* to copper deprivation, compensating for the deficiencies of Coxp activity but also for the loss of Sod1p activity by minimizing mitochondrial ROS (Broxton and Culotta, 2016).

**FIGURE 9:**
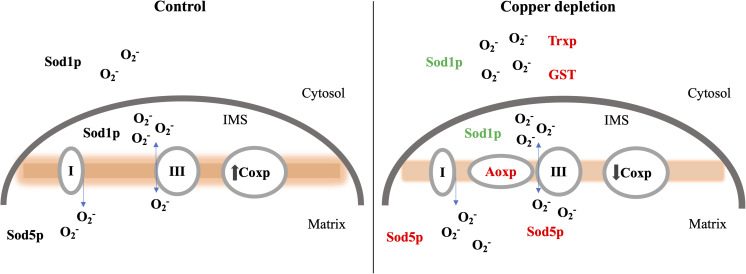
Changes in cytosolic and mitochondrial protein expression in response to copper deprivation. During copper deprivation the decrease of Sod1p in the cytosol and mitochondrial intermembrane space (IMS) as well as the decrease in cytochrome c oxidase (Cox1p) activity, promotes increase in the activity of the alternative oxidase enzyme (Aoxp) as well as an increase in superoxide dismutase Mn/Fe (Sod5p), thioredoxin (Trxp) and glutathione S transferase (GST). Proteins in red were increased; in green represents the protein with reduced abundance during copper deprivation.

We provide evidence that during copper deprivation in *P. brasiliensis* an alternative oxidase form of respiration is induced that is not coupled to ATP synthesis but maintains mitochondrial superoxide at low levels putatively compensating for decrease in Sod1p. Considering the low activity of Sod1p, both Sod5p and Aoxp may act to compensate for the loss of efficiency in the mitochondrial detoxification process and at the same time compensate the respiratory activity of the fungus. Further, thioredoxin and GST could compensate the decrease in Sod1 activity in the cytosol, a schematic drawing is depicted in Figure 9. Therefore, in the present study we proposes an adaptive response of *P. brasiliensis* to *in vitro* copper deprivation, a condition putatively faced by the fungus during infection, at least in macrophages. Metabolic shift toward beta-oxidation and glyoxylate cycle, ROS detoxification and respiration seem to be the most affected processes by the deprivation of this metal. In synthesis, copper deprivation compromised the activity of Sod1p, putatively affecting detoxification. This probably lead to increase in thioredoxin, GST, Sod5p, and Atx1p in addition, to up-regulation of the pentose phosphate pathway enzyme, 6-phosphogluconate dehydrogenase, related to the production of NADPH probably enhancing the response to the increase in ROS generated by copper deprivation.

## Data Availability Statement

The datasets presented in this study can be found in online repositories. The names of the repository/repositories and accession number(s) can be found at: http://www.peptideatlas.org/, PASS01563.

## Ethics Statement

The animal study was reviewed and approved by the Animal Use Ethics Committee of the Federal University of Goiás (CEUA-PRPI-UFG), under protocol number 092/17.

## Author Contributions

CA and GP conceived and designed the experiments. GP, JP, JC, CM, GT, and AC performed the experiments. GP, AB, WF, CR, MP, MS, and CA analyzed and interpreted the data. CA contributed to reagents and materials. GP, JC, and CA analyzed the data and wrote the manuscript. All authors contributed to the article and approved the submitted version.

## Conflict of Interest

The authors declare that the research was conducted in the absence of any commercial or financial relationships that could be construed as a potential conflict of interest.

## Footnotes

1 http://www.uniprot.org/

2 www.ncbi.nlm.nih.gov/genome/?term=Paracoccidioides

3 http://www.peptideatlas.org/

4 https://www.blast2go.com/

5 https://fungidb.org/fungidb/

6 http://www.genome.jp/kegg/

7 https://blast.ncbi.nlm.nih.gov/Blast.cgi

## Supplementary Material

FIGURE S1 Functional classification of differentially expressed proteins in yeast cells after 24 h of copper deprivation. All identified proteins were classified according to Functional Catalog (FunCat2). For the experiments the yeast cells were cultured in MMcM in the presence of copper (10 μM of CuSO_4_) or in the deprivation of this metal (50 μm BCS), afterward the proteins were extracted and analyzed by ITRAQ.

FIGURE S2 Obtaining the recombinant Atx1p. **(A)** PET32a:Atx1 vector. In red the *atx1* gene fused to the histidine tail (trxA-His6-atx1); black Ampicillin resistance gene (AmpR). **(B)** Standardization of induction of recombinant Atx1p with IPTG. M, molecular weight marker; T0, Control; T30, Induction time of 30 min; T1h, Induction time of 1 h; T2h, Induction time of 2 h; T3, Induction time of 3 h. **(C)** Purification of recombinant Atx1p. The recombinant protein was purified by nickel column affinity chromatography and the material was analyzed on 12% polyacrylamide gel. 1, Molecular weight marker; 2, Nickel resin after binding to recombinant Atx1p; 3, First wash with buffer containing 20 mM imidazole; 4, Second wash with buffer containing 20 mM imidazole; 5, Eluted fraction of recombinant Atx1p in buffer containing 250 mM imidazole. The arrow indicates the recombinant Atx1p. **(D)** Analysis of the reactivity of polyclonal anti-Atx1 antibodies by Western blotting. 1, Detection of Atx1p from *P. brasiliensis* yeast cells protein extract using anti-Atx1 antibodies; 2, Negative control – Reactivity of Atx1p with sera of pre-immune animals. The arrow indicates native Atx1p protein.

TABLE S1 Oligonucleotides employed in the experiments.

TABLE S2 *Paracoccidioides brasiliensis* proteins with increased abundance in yeast cells after 24 h of copper depletion.

TABLE S3 *Paracoccidioides brasiliensis* proteins with decreased abundance in yeast cells after 24 h of copper depletion.

## References

[B1] Araújo D. S. Pereira M. Portis I. G. De Castro Moreira Dos Santos A. Fontes W. De Sousa M. V. (2019). Metabolic peculiarities of *Paracoccidioides brasiliensis* dimorphism as demonstrated by iTRAQ labeling proteomics. *Front. Microbiol.* 10:555. 10.3389/fmicb.2019.00555 30949151 PMC6436475

[B2] Besold A. N. Gilston B. A. Radin J. N. Ramsoomair C. Culbertson E. M. Li C. X. (2018). Role of calprotectin in withholding zinc and copper from *Candida albicans*. *Infect. Immun.* 86 1–16. 10.1128/IAI.00779-17 29133349 PMC5778358

[B3] Bookout A. L. Cummins C. L. Kramer M. F. Pesola J. M. Mangelsdorf J. D. Mangelsdorf D. J. (2006). High-Throughput Real-Time Quantitative Reverse Transcription PCR. *Curr. Protoc. Mol. Biol.* 73 15.8.1–15.8.28. 10.1002/0471142727.mb1508s73 18265376

[B4] Borghouts C. Scheckhuber C. Q. Stephan O. Osiewacz H. D. (2002). Copper homeostasis and aging in the fungal model system *Podospora anserina*: differential expression of PaCtr3 encoding a copper transporter. *Int. J. Biochem. Cell Biol.* 34 1355–1371. 10.1016/S1357-2725(02)00078-X 12200031

[B5] Broxton C. N. Culotta V. C. (2016). An adaptation to low copper in *Candida albicans* involving SOD Enzymes and the alternative oxidase. *PLoS One* 11:e0168400. 10.1371/journal.pone.0168400 28033429 PMC5198983

[B6] Chaves E. G. A. Parente-Rocha J. A. Baeza L. C. Araújo D. S. Borges C. L. de Oliveira M. A. P. (2019). Proteomic analysis of *Paracoccidioides brasiliensis* during infection of alveolar macrophages primed or not by interferon-gamma. *Front. Microbiol.* 10:96. 10.3389/fmicb.2019.00096 30804901 PMC6371752

[B7] Dancis A. Haile D. Yuan D. S. Klausnert R. D. (1994). The *Saccharomyces cerevisiae* copper transport protein (Ctrlp). *J. Biol. Chem.* 269 25660–25667. 7929270

[B8] Dantas S. F. I. M. Vieira de Rezende T. C. Bailão A. M. Taborda C. P. da Silva Santos R. Pacheco de Castro K. (2009). Identification and characterization of antigenic proteins potentially expressed during the infectious process of *Paracoccidioides brasiliensis*. *Microbes Infect.* 11 895–903. 10.1016/j.micinf.2009.05.009 19500685

[B9] De Curcio J. S. Silva M. G. Silva Bailão M. G. Báo S. N. Casaletti L. Bailão A. M. (2017). Identification of membrane proteome of *Paracoccidioides lutzii* and its regulation by zinc. *Futur. Sci. OA* 3:FSO232. 10.4155/fsoa-2017-0044 29134119 PMC5676091

[B10] De Oliveira H. C. De Fátima Da Silva J. Matsumoto M. T. Marcos C. M. Peres Da Silva R. Moraes Da Silva R. A. (2014). Alterations of protein expression in conditions of copper-deprivation for *Paracoccidioides lutzii* in the presence of extracellular matrix components. *BMC Microbiol.* 14:302. 10.1186/s12866-014-0302-7 25609357 PMC4302596

[B11] Ding C. Festa R. A. Chen Y. Espart A. Palacios Ò Capdevila M. (2014a). *Cryptococcus neoformans* copper detoxification machinery is critical for fungal virulence. *Cell Host Microbe* 3 265–276. 10.1016/j.chom.2013.02.002 23498952 PMC3668348

[B12] Ding C. Festa R. A. Sun T. S. Wang Z. Y. (2014b). Iron and copper as virulence modulators in human fungal pathogens. *Mol. Microbiol.* 93 10–23. 10.1111/mmi.12653 24851950

[B13] Fukuoka M. Tokuda E. Nakagome K. Wu Z. Nagano I. Furukawa Y. (2017). *An Essential Role of N-terminal Domain of Copper Chaperone in the Enzymatic Activation of Cu/Zn-Superoxide Dismutase.* Amsterdam: Elsevier Inc. 10.1016/j.jinorgbio.2017.07.036 28780408

[B14] García-Santamarina S. Thiele D. J. (2015). Copper at the fungal pathogen-host axis. *J. Biol. Chem.* 290 18945–18953. 10.1074/jbc.R115.649129 26055724 PMC4521015

[B15] Georgatsou E. Mavrogiannis L. A. Fragiadakis G. S. Alexandraki D. (1997). The yeast Fre1p/Fre2p cupric reductases facilitate copper uptake and are regulated by the copper-modulated Mac1p activator. *J. Biol. Chem.* 272 13786–13792. 10.1074/jbc.272.21.13786 9153234

[B16] Gross C. Kelleher M. Iyer V. R. Brown P. O. Winge D. R. (2000). Identification of the copper regulon in *Saccharomyces cerevisiae* by DNA microarrays. *J. Biol. Chem.* 275 32310–32316. 10.1074/jbc.M005946200 10922376

[B17] Grossklaus D. A. Bailão A. M. Rezende T. C. V. Borges C. L. de Oliveira M. A. P. Parente J. A. (2013). Response to oxidative stress in *Paracoccidioides* yeast cells as determined by proteomic analysis. *Microbes Infect.* 15 347–364. 10.1016/j.micinf.2012.12.002 23421979

[B18] Hassett R. Kosman D. J. (1995). Evidence for Cu(II) reduction as a component of copper uptake by *Saccharomyces cerevisiae*. *J. Biol. Chem.* 270 128–134. 10.1074/jbc.270.1.128 7814363

[B19] Hernández O. Araque P. Tamayo D. Restrepo A. Herrera S. Mcewen J. G. (2015). Alternative oxidase plays an important role *Paracoccidioides brasiliensis* cellular homeostasis and morphological transition. *Med. Mycol.* 15 205–214. 10.1093/mmy/myu091 25631476

[B20] Horne J. T. Derek W. Hollomon P. M. W. (2001). Fungal respiration: a fusion of standard and alternative components. *Biochim. Biophys. Acta Bioenerg.* 1504 179–195. 10.1016/S0005-2728(00)00251-6 11245784

[B21] Horng Y. C. Cobine P. A. Maxfield A. B. Carr H. S. Winge D. R. (2004). Specific copper transfer from the Cox17 metallochaperone to both Sco1 and Cox11 in the assembly of yeast cytochrome c oxidase. *J. Biol. Chem.* 279 35334–35340. 10.1074/jbc.M404747200 15199057

[B22] Ikeda R. Sawamura K. (2008). Bacterial and H2O2 stress-induced apoptosis-like events in *Cryptococcus neoformans*. *Res. Microbiol.* 159 628–634. 10.1016/j.resmic.2008.07.006 18793720

[B23] Jungmann J. Reins H. A. Lee J. Romeo A. Hassett R. Kosman D. (1993). MAC1, a nuclear regulatory protein related to Cu-dependent transcription factors is involved in Cu/Fe utilization and stress resistance in yeast. *EMBO J.* 12 5051–5056. 10.1002/j.1460-2075.1993 8262047 PMC413765

[B24] Khalimonchuk O. Rödel G. (2005). Biogenesis of cytochrome c oxidase. *Mitochondrion* 5 363–388. 10.1016/j.mito.2005.08.002 16199211

[B25] Kim I. S. Sohn H. Y. Jin I. (2011). Adaptive stress response to menadione-induced oxidative stress in *Saccharomyces cerevisiae* KNU5377. *J. Microbiol.* 49 816–823. 10.1007/s12275-011-1154-6 22068500

[B26] Lacerda Pigosso L. Baeza L. C. Vieira Tomazett M. Batista Rodrigues Faleiro M. Brianezi Dignani de Moura V. M. Melo Bailão A. (2017). *Paracoccidioides brasiliensis* presents metabolic reprogramming and secretes a serine proteinase during murine infection. *Virulence* 8, 1417–1434. 10.1080/21505594.2017.1355660 28704618 PMC5711425

[B27] Ladomersky E. Khan A. Shanbhag V. Cavet J. S. Chan J. Weisman G. A. (2017). Host and pathogen copper-transporting P-Type ATPases function antagonistically during *Salmonella Infection*. *Infect. Immun.* 85 1–9. 10.1128/IAI.00351-17 28652309 PMC5563570

[B28] Lin S. J. Culotta V. C. (1995). The ATX1 gene of *Saccharomyces cerevisiae* encodes a small metal homeostasis factor that protects cells against reactive oxygen toxicity. *Proc. Natl. Acad. Sci. U.S.A.* 92 3784–3788. 10.1073/pnas.92.9.3784 7731983 PMC42046

[B29] Lin S. J. Pufahl R. A. Dancis A. O’Halloran T. V. Culotta V. C. (1997). A role for the *Saccharomyces cerevisiae* ATX1 gene in copper trafficking and iron transport. *J. Biol. Chem.* 272 9215–9220. 10.1074/jbc.272.14.9215 9083054

[B30] Mackie J. Szabo E. K. Urgast D. S. Ballou E. R. Childers D. S. MacCallum D. M. (2016). Host-imposed copper poisoning impacts fungal micronutrient acquisition during systemic *Candida albicans* infections. *PLoS One* 11:e0158683. 10.1371/journal.pone.0158683 27362522 PMC4928837

[B31] Martinez R. (2015). Epidemiology of Paracoccidioidomycosis. *Rev. do Inst. Med. Trop. São Paulo* 57(Suppl. 1), 11–20. 10.1590/S0036-46652015000700004 26465364 PMC4711199

[B32] McEwen J. G. Bedoya V. Patiño M. M. Salazar M. E. Restrepo A. (1987). Experimental murine paracoccidiodomycosis induced by the inhalation of conidia. *J. Med. Vet. Mycol.* 25 165–175. 10.1080/02681218780000231 3612432

[B33] Parente-Rocha J. A. Parente A. F. A. Baeza L. C. Bonfim S. M. R. C. Hernandez O. McEwen J. G. (2015). Macrophage interaction with *Paracoccidioides brasiliensis* yeast cells modulates fungal metabolism and generates a response to oxidative stress. *PLoS One* 10:e0137619. 10.1371/journal.pone.0137619 26360774 PMC4567264

[B34] Peña M. M. O. Koch K. A. Thiele D. J. (1998). Dynamic regulation of copper uptake and detoxification genes in *Saccharomyces cerevisiae*. *Mol. Cell. Biol.* 18 2514–2523. 10.1128/mcb.18.5.2514 9599102 PMC110631

[B35] Peña M. M. O. Lee J. Thiele D. J. (1999). A delicate balance: homeostatic control of copper uptake and distribution. *J. Nutr.* 129 1251–1260. 10.1093/jn/129.7.1251 10395584

[B36] Pena M. M. O. Puig S. Thiele D. J. (2000). Characterization of the *Saccharomyces cerevisiae* high affinity copper transporter Ctr3. *J. Biol. Chem.* 275 33244–33251. 10.1074/jbc.M005392200 10924521

[B37] Queiroz R. M. L. Charneau S. Mandacaru S. C. Schwämmle V. Lima B. D. Roepstorff P. (2014). Quantitative proteomic and phosphoproteomic analysis of *Trypanosoma cruzi* amastigogenesis. *Mol. Cell. Proteomics* 13 3457–3472. 10.1074/mcp.M114.040329 25225356 PMC4256497

[B38] Restrepo A. (1985). The ecology of *Paracoccidioides brasiliensis*: a puzzle still unsolved. *Sabouraudia* 23 323–334. 10.1080/00362178585380481 3906945

[B39] Restrepo A. Jimenez B. (1980). *Paracoccidioides brasiliensis*, growth of culture, yeast phase in a chemically defined. *J. Clin. Microbiol.* 12 279–281. 10.1128/jcm.12.2.279-281.1980 7229010 PMC273566

[B40] Ruppen I. Grau L. Orenes-Piñero E. Ashman K. Gil M. Algaba F. (2010). Differential protein expression profiling by iTRAQ-two-dimensional LC-MS/MS of human bladder cancer EJ138 cells transfected with the metastasis suppressor KiSS-1 gene. *Mol. Cell. Proteomics* 9 2276–2291. 10.1074/mcp.M900255-MCP200 20139371 PMC2953920

[B41] San-Blas G. NiÑo-Vega G. Iturriaga T. (2002). *Paracoccidioides brasiliensis* and paracoccidioidomycosis: molecular approaches to morphogenesis, diagnosis, epidemiology, taxonomy and genetics. *Med. Mycol.* 40 225–242. 10.1080/mmy.40.3.225.242 12146752

[B42] Silva M. G. Schrank A. Bailão E. F. L. C. Bailão A. M. Borges C. L. Staats C. C. (2011). The homeostasis of iron, copper, and zinc in *Paracoccidioides brasiliensis*, *Cryptococcus neoformans* var. *Grubii*, and *Cryptococcus gattii*: a comparative analysis. *Front. Microbiol.* 2:49. 10.3389/fmicb.2011.00049 21833306 PMC3153025

[B43] Singh R. Mailloux R. J. Puiseux-Dao S. Appanna V. D. (2007). Oxidative stress evokes a metabolic adaptation that favors increased NADPH synthesis and decreased NADH production in *Pseudomonas fluorescens*. *J. Bacteriol.* 189 6665–6675. 10.1128/JB.00555-07 17573472 PMC2045160

[B44] Smith A. D. Logeman B. L. Thiele D. J. (2017). Copper acquisition and utilization in fungi. *Physiol. Behav.* 176 139–148. 10.1016/j.physbeh.2017.03.040 28886682 PMC6827982

[B45] Song J. Li R. Jiang J. (2019). Copper homeostasis in *Aspergillus fumigatus*: opportunities for therapeutic development. *Front. Microbiol.* 10:774. 10.3389/fmicb.2019.00774 31031736 PMC6473158

[B46] Sugui J. A. Kim H. S. Zarember K. A. Chang Y. C. Gallin J. I. Nierman W. C. (2008). Genes differentially expressed in conidia and hyphae of *Aspergillus fumigatus* upon exposure to human neutrophils. *PLoS One* 3:e0002655. 10.1371/journal.pone.0002655 18648542 PMC2481287

[B47] Sun T. S. Ju X. Gao H. L. Wang T. Thiele D. J. Li J. Y. (2014). Reciprocal functions of *Cryptococcus neoformans* copper homeostasis machinery during pulmonary infection and meningoencephalitis. *Nat. Commun.* 5:5550. 10.1038/ncomms6550 25417972

[B48] Tamayo D. Muñoz J. F. Lopez Á Urán M. Herrera J. Borges C. L. (2016). Identification and analysis of the role of superoxide dismutases isoforms in the pathogenesis of *Paracoccidioides* spp. *PLoS Negl. Trop. Dis.* 10:e0004481. 10.1371/journal.pntd.0004481 26963091 PMC4786090

[B49] Urbanski N. K. Berêsewicz A. (2000). Generation of. OH initiated by interaction of Fe2+ and Cu+ with dioxygen; comparison with the Fenton chemistry. *Acta Biochim. Pol.* 47 951–962. 10.18388/abp.2000_3950 11996118

[B50] Vishwakarma A. Tetali S. D. Selinski J. Scheibe R. Padmasree K. (2015). Importance of the alternative oxidase (AOX) pathway in regulating cellular redox and ROS homeostasis to optimize photosynthesis during restriction of the cytochrome oxidase pathway in *Arabidopsis thaliana*. *Ann. Bot.* 116 555–569. 10.1093/aob/mcv122 26292995 PMC4578005

[B51] Waterman S. R. Park Y. D. Raja M. Qiu J. Hammoud D. A. O’Halloran T. V. (2012). Role of CTR4 in the virulence of *Cryptococcus neoformans*. *mBio* 3:e00285-12. 10.1128/mBio.00285-12 23033470 PMC3518914

[B52] Weissman Z. Berdicevsky I. Cavari B. Z. Kornitzer D. (2000). The high copper tolerance of *Candida albicans* is mediated by a P-type ATPase. *Proc. Natl. Acad. Sci. U.S.A.* 97 3520–3525. 10.1073/pnas.97.7.3520 10737803 PMC16272

[B53] White C. Lee J. Kambe T. Fritsche K. Petris M. J. (2009). A role for the ATP7A copper-transporting ATPase in macrophage bactericidal activity. *J. Biol. Chem.* 284 33949–33956. 10.1074/jbc.M109.070201 19808669 PMC2797165

[B54] Wiemann P. Perevitsky A. Lim F. Y. Shadkchan Y. Knox B. P. Landero Figueora J. A. (2017). *Aspergillus fumigatus* copper export machinery and reactive oxygen intermediate defense counter host copper-mediated oxidative antimicrobial offense. *Cell Rep.* 19 1008–1021. 10.1016/j.celrep.2017.04.019 28467895 PMC5512462

[B55] Zhu Z. Labbé S. Peña M. M. O. Thiele D. J. (1998). Copper differentially regulates the activity and degradation of yeast Mac1 transcription factor. *J. Biol. Chem.* 273 1277–1280. 10.1074/jbc.273.3.1277 9430656

