# Supplementary figures and images for: Metabolic Adaptation of Paracoccidioides brasiliensis in Response to in vitro Copper Deprivation
Source: Front Microbiol. 2020 Aug 10;11:1834. doi: 10.3389/fmicb.2020.01834 (PMC7430155; doi:10.3389/fmicb.2020.01834)

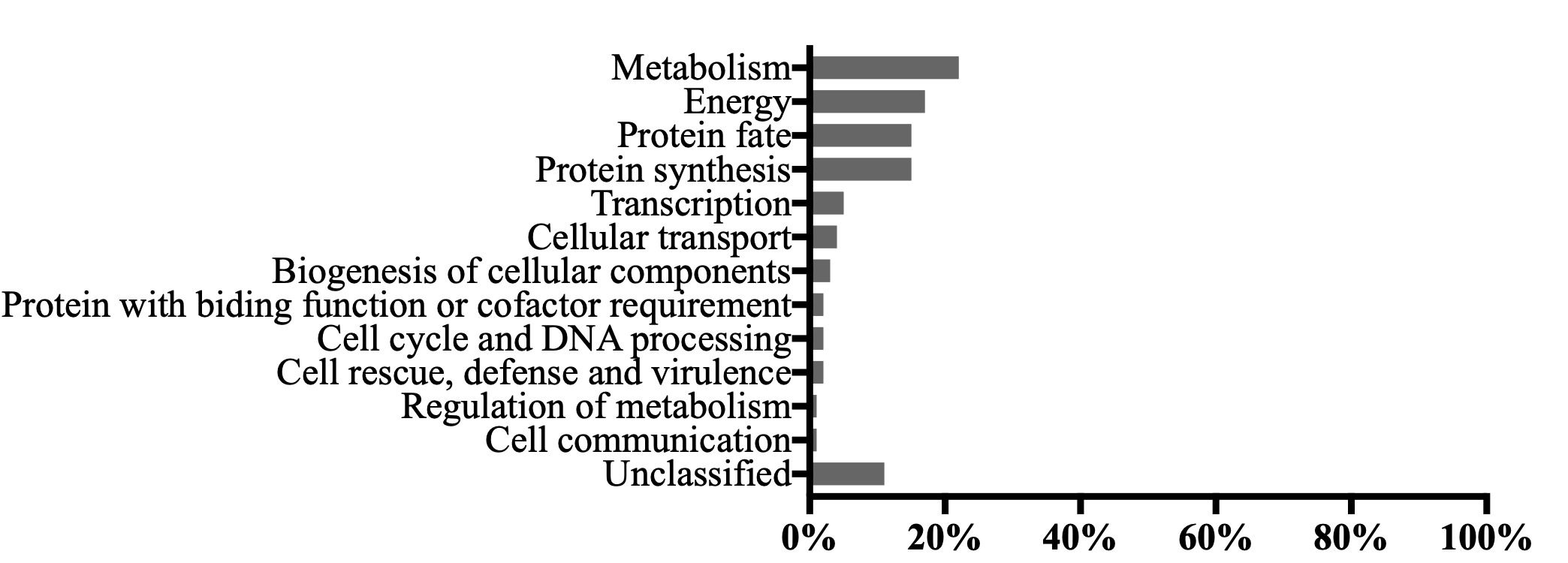

Supplement: FIGURE S1 — Functional classification of differentially expressed proteins in yeast cells after 24 h of copper deprivation. All identified proteins were classified according to Functional Catalog (FunCat2). For the experiments the yeast cells were cultured in MMcM in the presence of copper (10 μM of CuSO4) or in the deprivation of this metal (50 μm BCS), afterward the proteins were extracted and analyzed by ITRAQ. [file Image_1.TIFF]

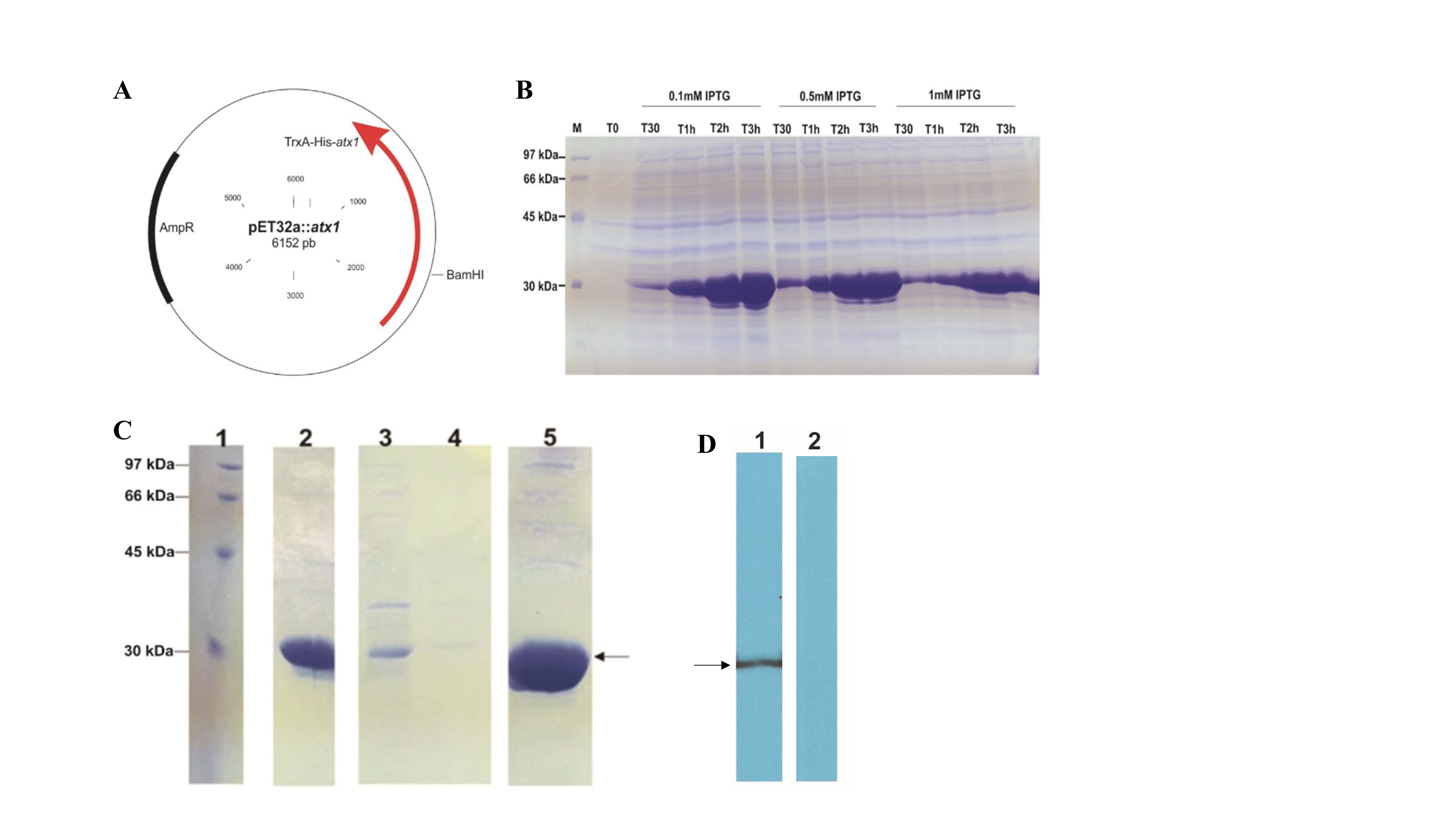

Supplement: FIGURE S2 — Obtaining the recombinant Atx1p. (A) PET32a:Atx1 vector. In red the atx1 gene fused to the histidine tail (trxA-His6-atx1); black Ampicillin resistance gene (AmpR). (B) Standardization of induction of recombinant Atx1p with IPTG. M, molecular weight marker; T0, Control; T30, Induction time of 30 min; T1h, Induction time of 1 h; T2h, Induction time of 2 h; T3, Induction time of 3 h. (C) Purification of recombinant Atx1p. The recombinant protein was purified by nickel column affinity chromatography and the material was analyzed on 12% polyacrylamide gel. 1, Molecular weight marker; 2, Nickel resin after binding to recombinant Atx1p; 3, First wash with buffer containing 20 mM imidazole; 4, Second wash with buffer containing 20 mM imidazole; 5, Eluted fraction of recombinant Atx1p in buffer containing 250 mM imidazole. The arrow indicates the recombinant Atx1p. (D) Analysis of the reactivity of polyclonal anti-Atx1 antibodies by Western blotting. 1, Detection of Atx1p from P. brasiliensis yeast cells protein extract using anti-Atx1 antibodies; 2, Negative control – Reactivity of Atx1p with sera of pre-immune animals. The arrow indicates native Atx1p protein. [file Image_2.TIF]
