# Supplementary material for: Metabolic Adaptation of Paracoccidioides brasiliensis in Response to in vitro Copper Deprivation
Source: Front Microbiol. 2020 Aug 10;11:1834. doi: 10.3389/fmicb.2020.01834 (PMC7430155; doi:10.3389/fmicb.2020.01834)
Supplement: TABLE S1 — Oligonucleotides employed in the experiments. [file Table_1.docx]

**Supplementary Table 1** – Oligonucleotides employed in the experiments.

| **Gene/Accession Numbers** | **Oligonucleotides/Gene ID** | **Tm (^0^C)** |
| --- | --- | --- |
| L34 Fwd/PADG_04085 | 5’TCAATCTCTCCCGCGAATCC3’ | 62 |
| L34 Rev/ PADG_04085 | 5’AGTTGGCGATTGTTGTGCGG3’ | 62 |
|  |  |  |
| CTR3 Fwd/ PADG_05084 | 5’GCTCCTGCGTGATTTCTATGC3’ | 60 |
| CTR3 Rev/ PADG_05084 | 5’CTCAAGCGTTTCCCAGCGAG3’ | 60 |
|  |  |  |
| ATX1 Fwd/ PADG_02352 | 5’GGCTGATATCGGATCCATGGCTTCCGTCGAACATC3’ | 56 |
| ATX1 Rev/ PADG_02352 | 5’GTGCGGCCGCAAGCTTTCAAACATCCTTCGGCTCGO3’ | 56 |
| α-TUBULIN TaqMan | 140936 | - |
| ATP7a TaqMan | 538 | - |

(-) No information provided.
