# Supplementary material for: Metabolic Adaptation of Paracoccidioides brasiliensis in Response to in vitro Copper Deprivation
Source: Front Microbiol. 2020 Aug 10;11:1834. doi: 10.3389/fmicb.2020.01834 (PMC7430155; doi:10.3389/fmicb.2020.01834)
Supplement: TABLE S2 — Paracoccidioides brasiliensis proteins with increased abundance in yeast cells after 24 h of copper depletion. [file Table_2.docx]

**Supplementary Table 2** - *Paracoccidioides brasiliensis* proteins with increased abundance in yeast cells after 24 h of copper depletion.

| **GenBank Accession Number ^a^** | | **Description^b^** | **Unique peptides^c^** |  | **Fold^d^** | **Score^e^** |
| --- | --- | --- | --- | --- | --- | --- |
|  | | **Metabolism** |  |  |  |  |
|  | ***Amino acid metabolism*** | | | | |  |
| PADG_02214 | | 4-aminobutyrate aminotransferase | 15 |  | 1,55 | 12,59 |
| PADG_01797 | | Dihydrolipoamide S-acetyltransferase | 2 |  | 1,49 | 34,726 |
| PADG_03514 | | 2-oxoisovalerate dehydrogenase subunit alpha | 5 |  | 1,44 | 22,906 |
| PADG_03627 | | 2-oxoisovalerate dehydrogenase subunit beta | 7 |  | 1,40 | 15,386 |
| PADG_07369 | | Isovaleryl-CoA dehydrogenase | 13 |  | 1,58 | 12,192 |
| PADG_01621 | | Aspartate aminotransferase | 23 |  | 1,41 | 34,916 |
| PADG_04516 | | Glutamate dehydrogenase | 3 |  | 1,23 | 9,571 |
| PADG_00210 | | Glycine dehydrogenase | 20 |  | 1,63 | 11,515 |
| PADG_08465 | | Fumarylacetoacetase | 9 |  | 1,45 | 45,951 |
| PADG_08466 | | Homogentisate 1,2-dioxygenase | 13 |  | 1,44 | 9,775 |
| PADG_00663 | | Homoserine dehydrogenase | 6 |  | 1,44 | 14,475 |
| PADG_08406 | | *O*-acetylhomoserine (thiol)-lyase | 14 |  | 1,68 | 9,536 |
| PADG_01928 | | *S*-adenosylmethionine synthetase | 7 |  | 1,65 | 14,493 |
| PADG_06252 | | 1,2-dihydroxy-3-keto-5-methylthiopentenedioxygenase | 4 |  | 1,76 | 12,596 |
| PADG_06289 | | Lysine decarboxylase | 3 |  | 1,29 | 16,68 |
| PADG_00405 | | Choline dehydrogenase | 4 |  | 1,63 | 15,384 |
| PADG_06252 | | 1,2-dihydroxy-3-keto-5-methylthiopentenedioxygenase | 4 |  | 1,76 | 15,564 |
| PADG_07241 | | Dihydroxy-acid dehydratase | 6 |  | 1,40 | 20,631 |
|  | ***Nitrogen, sulfur and selenium metabolism*** | | | | |  |
| PADG_02048 | | Nitroredutase | 5 |  | 1,52 | 4,521 |
| PADG_06490 | | Formamidase | 14 |  | 1,40 | 52,093 |
| PADG_00637 | | Arginase | 8 |  | 1,57 | 29,428 |
| PADG_08300 | | ornithine transcarbamilase | 6 |  | 1,48 | 20,304 |
| ***Nucleotide, nucleoside, nucleobase metabolism*** | | |  |  |  |  |
| PADG_00322 | | Xantina-fosforibosil-transferase | 5 |  | 1,36 | 74,748 |
|  | ***Phosphate metabolism*** | | | | |  |
| PADG_04175 | | Inorganic pyrophosphatase | 2 |  | 1,29 | 10,732 |
|  | ***Biosynthesis of vitamins, cofactors and prosthetics groups*** | | | | |  |
| PADG_01886 | | Adenosyl homocysteinase | 23 |  | 1,53 | 10,582 |
| PADG_05822 | | Pyridoxine biosynthesis | 4 |  | 1,45 | 75,16 |
|  | ***C-compound and carbohydrate metabolism*** | | | | |  |
| PADG_01486 | | Short chain dehydrogenase/reductase family | 2 |  | 1,29 | 22,341 |
| PADG_00604 | | Phosphoacetylglucosamine mutase | 3 |  | 1,56 | 108,085 |
| PADG_03671 | | Phenylpyruvate tautomerase | 4 |  | 1,55 | 28,847 |
| PADG_06199 | | FAD binding domain-containing protein | 5 |  | 1,48 | 30,384 |
|  | ***Lipid, fatty acid, and isoprenoid metabolism*** | | | | |  |
| PADG_00254 | | Fatty acid synthase subunit alpha reductase | 32 |  | 1,40 | 9,621 |
| PADG_05904 | | Dihydrolipoamide succinyltransferase | 7 |  | 1,48 | 9,486 |
| PADG_00678 | | Oxiterol protein |  |  |  |  |
|  | ***Metabolism of vitamins, cofactors, and protetics group*** | | | | |  |
| PADG_00443 | | Dihydropteroate synthase | 10 |  | 1,60 | 45,283 |
| PADG_00607 | | Riboflavin synthase alpha chain | 2 |  | 1,48 | 20,304 |
|  | **Energy** | | | | |  |
|  | ***Glycolysis and gluconeogenesis*** | | | | |  |
| PADG_00852 | | Fructose 1,6, biphosphate aldolase | 2 |  | 1,64 | 5,938 |
| PADG_05109 | | Phosphoenolpyruvate carboxykinase | 20 |  | 1,52 | 54,408 |
| PADG_01706 | | Fructose-1,6-bisphosphatase | 7 |  | 2,57 | 28,593 |
| PADG_02411 | | Glyceraldehyde-3-phosphate dehydrogenase | 26 |  | 1,31 | 51,116 |
| PADG_05081 | | Aldehyde dehydrogenase | 14 |  | 1,52 | 54,187 |
|  | ***Fermentation*** | | | | |  |
| PADG_00714 | | Pyruvate decarboxylase | 15 |  | 2,56 | 64,219 |
|  | ***Glyoxylate cycle*** | | | | |  |
| PADG_04709 | | Isocitrate lyase | 9 |  | 1,43 | 23,482 |
| PADG_11845 | | Aconitate hydratase | 37 |  | 1,32 | 14,452 |
| PADG_08059 | | Malate dehydrogenase | 16 |  | 1,65 | 12,985 |
|  | ***Citric acid cycle*** | | | | |  |
| PADG_04249 | | Isocitrate dehydrogenase | 16 |  | 1,60 | 12,989 |
| PADG_06494 | | Dihydrolipoyl dehydrogenase | 18 |  | 1,42 | 44,83 |
|  | ***Pentose-phosphate pathway*** | | | | |  |
| PADG_03651 | | 6-phosphogluconate dehydrogenase | 15 |  | 1,54 | 9,235 |
| PADG_04604 | | Transketolase | 17 |  | 1,50 | 40,523 |
|  | ***Electron transport and membrane-associated energy conservation*** | | | | |  |
| PADG_11468 | | NADPH-dependent diflavin oxidoreductase 1 | 3 |  | 1,29 | 32,277 |
| PADG_05523 | | Quinone oxidoreductase | 4 |  | 1,46 | 33,41 |
| PADG_11302 | | VosA protein | 2 |  | 1,48 | 28,593 |
| ***Respiration*** | | | |  |  |  |
| PADG_05905 | | NADH dehydrogenase | 3 |  | 1,51 | 132,419 |
| PADG_11605 | | NADPH-cytochrome P450 reductase | 13 |  | 1,67 | 48,746 |
| PADG_08349 | | ATP synthase beta subunit | 23 |  | 1,45 | 43,125 |
| PADG_07081 | | Electron transfer flavoprotein subunit alpha | 4 |  | 1,59 | 5,701 |
| PADG_03747 | | Alternative oxidase | 2 |  | 2,37 | 48,917 |
| PADG_03559 | | Cytochrome b5 | 3 |  | 1,35 | 54,408 |
| ***Oxidation of fatty acids*** | | | |  |  |  |
| PADG_00382 | | Acetyl-CoA acyltransferase | 16 |  | 1,50 | 12,314 |
| PADG_01687 | | Acetyl-CoA acyltransferase | 18 |  | 1,50 | 54,56 |
| PADG_02852 | | Acyl-CoA dehydrogenase | 12 |  | 1,57 | 23,975 |
| PADG_07023 | | Carnitine O-acetyltransferase | 9 |  | 1,49 | 45,439 |
|  | **Cell cycle and DNA processing** | | | | |  |
|  | ***DNA processing*** | | | | |  |
| PADG_05798 | | Single-strand binding protein family | 8 |  | 1,51 | 12,998 |
| PADG_02683 | | UV excision repair protein Rad23 | 4 |  | 1,50 | 9,479 |
| PADG_00656 | | [Non-histone chromosomal protein 6](https://www.ncbi.nlm.nih.gov/protein/EEH44367.2) | 3 |  | 1,57 | 8,972 |
|  | **Transcription** | | | | |  |
|  | ***mRNA synthesis*** | | | | |  |
| PADG_02555 | | Nucleic acid-binding protein | 14 |  | 1,51 | 65,114 |
| PADG_08717 | | Splicing factor 3B subunit 4 | 18 |  | 1,41 | 40,476 |
| PADG_05190 | | Coativador RNA polimerase II | 2 |  | 1,37 | 24,671 |
|  | ***Transcriptional control*** | | | | |  |
| PADG_00873 | | Histone H3 | 5 |  | 1,33 | 29,329 |
| PADG_05906 | | Histone H2A | 2 |  | 1,67 | 5,864 |
| PADG_02410 | | Histone deacetylase hda1 | 2 |  | 1,39 | 5,938 |
|  | ***Transcription repression*** | | | | |  |
| PADG_06182 | | Transcriptional repressor TUP1 | 7 |  | 1,46 | 25,825 |
|  | ***RNA processing*** | | | | |  |
| PADG_00041 | | Ran gtpase-activating protein | 4 |  | 1,40 | 11,807 |
| PADG_05393 | | mRNA decapping hydrolase | 2 |  | 1,38 | 10,141 |
|  | **Protein synthesis** | | | | |  |
|  | ***Ribosome biogenesis*** | | | | |  |
| PADG_01407 | | 40S ribosomal protein | 21 |  | 1,70 | 30,995 |
| PADG_00335 | | 40S ribosomal protein S14 | 10 |  | 1,45 | 20,335 |
| PADG_04118 | | 60S ribosomal protein L38 | 4 |  | 1,80 | 43,681 |
| PADG_06680 | | 40S ribosomal protein S22 | 7 |  | 1,54 | 14,578 |
| PADG_07863 | | 40S ribosomal protein S8 | 6 |  | 1,68 | 2,121 |
| PADG_04315 | | 40S ribosomal protein L24 | 10 |  | 1,63 | 6,078 |
| PADG_12365 | | 40S ribosomal protein S8 | 8 |  | 1,66 | 6,114 |
| PADG_02828 | | 60S ribosomal protein l10a | 8 |  | 1,50 | 23,192 |
| PADG_02888 | | 60S ribosomal protein L6 | 10 |  | 1,46 | 14,08 |
| PADG_03778 | | 60S ribosomal protein L10-A | 9 |  | 1,58 | 12,002 |
| PADG_03873 | | 60S ribosomal protein L20 | 8 |  | 1,57 | 9,089 |
| PADG_04065 | | 60S ribosomal protein L36 | 4 |  | 1,48 | 23,125 |
| PADG_04402 | | 60S ribosomal protein L34-A | 3 |  | 1,66 | 32,009 |
| PADG_12253 | | 60S ribosomal protein L3 | 20 |  | 1,55 | 47,14 |
| PADG_04449 | | 60S ribosomal protein l23e | 5 |  | 1,47 | 10,876 |
| PADG_05338 | | 60S ribosomal protein L18 | 7 |  | 1,51 | 7,342 |
| PADG_05883 | | 60S ribosomal protein l23a | 7 |  | 1,52 | 15,12 |
| PADG_06568 | | 60S ribosomal protein L23 | 8 |  | 1,32 | 3,456 |
| PADG_08213 | | 40S ribosomal protein S0 | 6 |  | 1,60 | 32,29 |
| PADG_07864 | | 40S ribosomal protein S8 | 6 |  | 1,68 | 17,10 |
| ***Translation*** | | | |  |  |  |
| PADG_6265 | | Elongation factor 1-gamma | 28 |  | 1,45 | 6,845 |
| PADG_8125 | | Elongation fator 2 | 36 |  | 1,70 | 3,224 |
|  | ***Aminoacyl-tRNA-synthetases*** | | | | |  |
| PADG_01558 | | Histidyl-tRNA synthetase | 11 |  | 1,64 | 5,246 |
| PADG_02918 | | Prolyl-tRNA synthetase | 4 |  | 1,40 | 6,481 |
| PADG_03440 | | Prolyl-tRNA synthetase | 6 |  | 1,37 | 14,578 |
|  | **Protein fate** | | | | |  |
|  | ***Protein folding and stabilization*** | | | | |  |
| PADG_00001 | | Peptidyl-prolyl cis-trans isomerase H | 6 |  | 1,63 | 21,878 |
| PADG_00207 | | Hsp40 Heat shock protein | 4 |  | 1,77 | 13,174 |
| PADG_00430 | | Hsp7 protein | 35 |  | 1,57 | 2,738 |
| PADG_03562 | | Hsp70 protein | 32 |  | 1,44 | 35,185 |
| PADG_05094 | | T-complex protein 1 subunit zeta | 38 |  | 1,54 | 34,681 |
| PADG_08369 | | Hsp60-like protein | 43 |  | 1,54 | 27,583 |
|  | ***Protein targeting, sorting and translocation*** | | | | |  |
| PADG_03882 | | Mitochondrial import inner membrane translocase | 3 |  | 1,40 | 29,394 |
|  | ***Protein modification*** | | | | |  |
| PADG_02637 | | Ubiquitin conjugating enzyme | 4 |  | 1,59 | 14,332 |
| ***Modification by phosphorylation, dephosphorylation, autophosphorylation*** | | | |  |  |  |
| PADG_02212 | | Serine/threonine-protein phosphatase | 5 |  | 1,34 | 3,951 |
| PADG_07925 | | Ubiquitin-conjugating enzyme | 2 |  | 1,65 | 3,191 |
| PADG_05245 | | Ubiquitin-NEDD8-like protein RUB2 | 2 |  | 1,62 | 31,053 |
|  | ***Protein processing (proteolytic)*** | | | | |  |
| PADG_01992 | | Mitochondrial-processing peptidase subunit alpha | 2 |  | 1,45 | 26,413 |
| PADG_06766 | | Mitochondrial-processing peptidase subunit beta | 8 |  | 1,35 | 12,076 |
|  | ***Assembly of protein complex*** | | | | |  |
| PADG_05335 | | Iron sulfur cluster assembly protein | 5 |  | 1,44 | 3,13 |
| PADG_07964 | | Vacuolar ATP synthase subunit E | 4 |  | 1,56 | 22,858 |
|  | ***Protein/peptide degradation*** | | | | |  |
| PADG_04167 | | Aspartyl aminopeptidase | 10 |  | 1,57 | 2,715 |
| PADG_05922 | | Cytosolic non-specific dipeptidase | 10 |  | 1,54 | 6,216 |
| PADG_06290 | | Proteossome endopeptidase complex | 4 |  | 1,48 | 2,953 |
|  | ***Cytoplasmic and nuclear protein degradation*** | | | | |  |
| PADG_08442 | | Proteasome component Y13 | 8 |  | 1,37 | 16,991 |
|  | ***Proteasomal degradation (ubiquitin/proteasomal pathway)*** | | | | |  |
| PADG_02735 | | Proteasome component PRE6 | 4 |  | 1,38 | 9,734 |
| PADG_03680 | | Proteasome component PRE2 | 6 |  | 1,51 | 11,433 |
| PADG_03982 | | Proteasome component C1 | 12 |  | 1,40 | 2,715 |
|  | **Protein with biding function or cofator requirement** | | | | |  |
|  | ***DNA binding*** | | | | |  |
| PADG_04311 | | Cellular nucleic acid-binding protein | 5 |  | 1,39 | 21,911 |
|  | ***RNA binding*** | | | | |  |
| PADG_07884 | | Polyadenylate-binding protein | 21 |  | 1,39 | 4,573 |
|  | ***GTP binding*** | | | | |  |
| PADG_04048 | | Small COPII coat GTPase sar1 | 2 |  | 1,74 | 35,844 |
|  | **Regulation of metabolism** | | | | |  |
|  | ***Enzyme inhibitor*** | | | | |  |
| PADG_01891 | | Translation initiation factor RLI1 | 6 |  | 1,72 | 15,238 |
|  | **Cellular transport** | | | | |  |
| PADG_00044 | | Nucleolin (492 a | 2 |  | 1,38 | 6,188 |
| PADG_01847 | | Nucleolin (452 aa) | 3 |  | 1,42 | 8,972 |
| PADG_02352 | | Copper chaperone ATX1 | 2 |  | 1,86 | 16,890 |
| PADG_03562 | | Chaperone Dnak | 34 |  | 1,66 | 12,345 |
| PADG_07014 | | Vesicular-fusion protein sec17 | 5 |  | 1,47 | 10,029 |
|  | **Cell communication** | | | | |  |
|  | ***Cellular signaling*** | | | | |  |
| PADG_01530 | | G-protein comlpex beta subunit cpcb |  |  |  |  |
|  | **Cell rescue, defence and virulence** | | | | |  |
|  | ***Oxidative stress response*** | | | | |  |
| PADG_05504 | | Thioredoxin | 8 |  | 1,59 | 39,381 |
| PADG_01954 | | Superoxide dismutase Mn/Fe dependente | 7 |  | 1,86 | 20,034 |
| PADG_02526 | | Glutathione S transferase | 2 |  | 1,83 | 4,573 |
|  | **Biogenesis of cellular componentes** | | | | |  |
|  | ***Cell wall*** | | | | |  |
| PADG_04312 | | UDP-N-acetylglucosamine pyrophosphorylase | 6 |  | 1,38 | 12,989 |
| PADG_00912 | | UDP-galactopyranose mutase | 12 |  | 1,49 | 7,898 |
| PADG_12426 | | 1,4-alpha-glucan-branching enzyme | 5 |  | 1,48 | 3,454 |
|  | ***Actin cytoskeleton*** | | | | |  |
| PADG_05538 | | Actin | 4 |  | 1,42 | 35,594 |
| PADG_12077 | | Actin | 11 |  | 1,43 | 37,732 |
|  | **Unclassified** | | | | |  |
| PADG_00440 | | Hypothetical protein | 6 |  | 1,55 | 12,52 |
| PADG_00676 | | Hypothetical protein | 5 |  | 1,37 | 8,788 |
| PADG_01343 | | Hypothetical protein | 6 |  | 1,55 | 2,367 |
| PADG_02342 | | Hypothetical protein | 7 |  | 1,60 | 9,589 |
| PADG_01867 | | Hypothetical protein | 12 |  | 1,67 | 6,895 |
| PADG_03203 | | Hypothetical protein | 7 |  | 1,47 | 7,126 |
| PADG_03244 | | Hypothetical protein | 3 |  | 1,34 | 17,104 |
| PADG_04439 | | Hypothetical protein | 6 |  | 1,59 | 13,792 |
| PADG_04636 | | Hypothetical protein | 11 |  | 1,45 | 18,998 |
| PADG_06488 | | Hypothetical protein | 19 |  | 1,46 | 30,762 |
| PADG_07264 | | Hypothetical protein | 3 |  | 1,50 | 43,141 |
| PADG_07627 | | Hypothetical protein | 2 |  | 1,43 | 33,464 |
| PADG_07670 | | Hypothetical protein | 3 |  | 1,39 | 30,041 |
| PADG_07836 | | Hypothetical protein | 5 |  | 2,79 | 35,185 |
| PADG_08037 | | Hypothetical protein | 2 |  | 1,48 | 15,238 |
| PADG_08715 | | Hypothetical protein | 9 |  | 1,59 | 15,164 |
| PADG_02981 | | Hypothetical protein | 4 |  | 1,29 | 11,319 |

^a,b)^ Accession Number and description of protein according to GenBank NCBI and MIPS database respectively:

GenBank NCBI: https://www.ncbi.nlm.nih.gov/genbank/ and Pedant on MIPS-Functional Catalogue (http://pedant.helmholtz-muenchen.de/);^c, d)^ indicates the number of peptides identified for a given protein and the fold change value, ^e)^ Sum PEP Score: Posterior error probability (PEP) values of the peptide spectrum matches (PSMs).
