# Supplementary material for: Metabolic Adaptation of Paracoccidioides brasiliensis in Response to in vitro Copper Deprivation
Source: Front Microbiol. 2020 Aug 10;11:1834. doi: 10.3389/fmicb.2020.01834 (PMC7430155; doi:10.3389/fmicb.2020.01834)
Supplement: TABLE S3 — Paracoccidioides brasiliensis proteins with decreased abundance in yeast cells after 24 h of copper depletion. [file Table_3.docx]

^v^ **Supplementary Table 3 -** *Paracoccidioides brasiliensis* proteins with decreased abundance in yeast cells after 24 h of copper depletion.

| **GenBank Accession Number ^a^** | **Description^b^** | **Unique peptides^c^** | **Fold^d^** | **Score^e^** |
| --- | --- | --- | --- | --- |
|  | **Metabolism** |  |  |  |
| [PADG_06332](http://www.ncbi.nlm.nih.gov/sites/entrez?db=gene&term=PADG_06332) | Pro-apoptotic serine protease | 2 | 0,78 | 12,776 |
| PADG_04293 | [adenine phosphoribosyltransferase](https://www.ncbi.nlm.nih.gov/protein/XP_010759790.1) | 3 | 0,83 | 9,265 |
|  | **Energy** |  |  |  |
| PADG_12145 | Uracil-regulated protein 1 | 4 | 0,81 | 3,341 |
| PADG_12144 | GTP cyclohydrolase | 2 | 0,77 | 21,078 |
|  | **Protein fate** |  |  |  |
| [PADG_07190](http://www.ncbi.nlm.nih.gov/sites/entrez?db=gene&term=PADG_07190) | Proteasome endopeptidase complex | 3 | 0,79 | 16,155 |
| PADG_02212 | Serine/threonine-protein phosphatase | 4 | 0,80 | 6,019 |
|  | **Cellular transport** |  |  |  |
| PADG_07804 | Protein transporter SEC23 | 2 | 0,83 | 8,991 |
|  | **Cell rescue, defence and virulence** |  |  |  |
| PADG_07418 | Superoxide dismutase Cu-Zn | 5 | 0,63 | 20,033 |
|  | **Unclassified** |  |  |  |
| [PADG_01900](http://www.ncbi.nlm.nih.gov/sites/entrez?db=gene&term=PADG_01900) | Hypothetical protein | 3 | 0,78 | 14,818 |

^a,b)^ Accession Number and description of protein according to Genbank NCBI and MIPS database respectively:

Genbank NCBI: https://www.ncbi.nlm.nih.gov/genbank/ and Pedant on MIPS-Functional Catalogue (http://pedant.helmholtz-muenchen.de/); ^c, d)^ indicates the number of peptides identified for a given protein and the fold change value, ^e)^ Sum PEP Score: Posterior error probability (PEP) values of the peptide spectrum matches (PSMs).
